# Supplementary material for: Designer Sinorhizobium meliloti strains and multi-functional vectors enable direct inter-kingdom DNA transfer
Source: PLoS One. 2019 Jun 17;14(6):e0206781. doi: 10.1371/journal.pone.0206781 (PMC6576745; doi:10.1371/journal.pone.0206781)
Supplement: S1 File — Figure A. Verification of hsdR deletion in reduced S. meliloti strains by diagnostic PCR. Figure B. Diagnostic restriction digest of pAGE and pBGE vectors. Figure C. Vector stability assay of pAGE2.0 in S. meliloti over 50 generations. Figure D. Workflow of optimized electroporation transformation protocol for S. meliloti. Figure E. Workflow of optimized PEG-mediated transformation protocol for S. meliloti. Figure F. PEG-mediated transformation of pAGE vectors into S. meliloti. Figure G. EcoRV-HF diagnostic digest of pAGE1.0 vectors extracted from 20 E. coli colonies following conjugation from S. meliloti to E. coli. Figure H. EcoRV-HF diagnostic digest of pAGE1.0 vectors extracted from 20 E. coli colonies following conjugation from S. meliloti to P. tricornutum. Figure I. EcoRV-HF diagnostic digest of pAGE1.0 vectors extracted from 20 E. coli colonies following conjugation from S. meliloti to S. cerevisiae. (DOCX) [file pone.0206781.s001.docx]

**Designer *Sinorhizobium meliloti* strains and multi-functional vectors enable direct inter-kingdom DNA transfer**

Stephanie L. Brumwell^1^, Michael R. MacLeod^2^, Tony Huang^1^, Ryan R. Cochrane^1^, Rebecca S. Meaney^3^, Maryam Zamani^2^, Ola Matysiakiewicz^4^, Kaitlyn N. Dan^1^, Preetam Janakirama^3^, David R. Edgell^1^, Trevor C. Charles^4^, Turlough M. Finan^2^, Bogumil J. Karas^1,3*^

^1^Department of Biochemistry, Schulich School of Medicine and Dentistry, Western University, London, ON, Canada

^2^Department of Biology, McMaster University, Hamilton, ON, Canada

^3^Designer Microbes Inc., London, ON, Canada

^4^Department of Biology, University of Waterloo, Waterloo, ON, Canada

Corresponding author:

Bogumil J. Karas: bkaras@uwo.ca

**
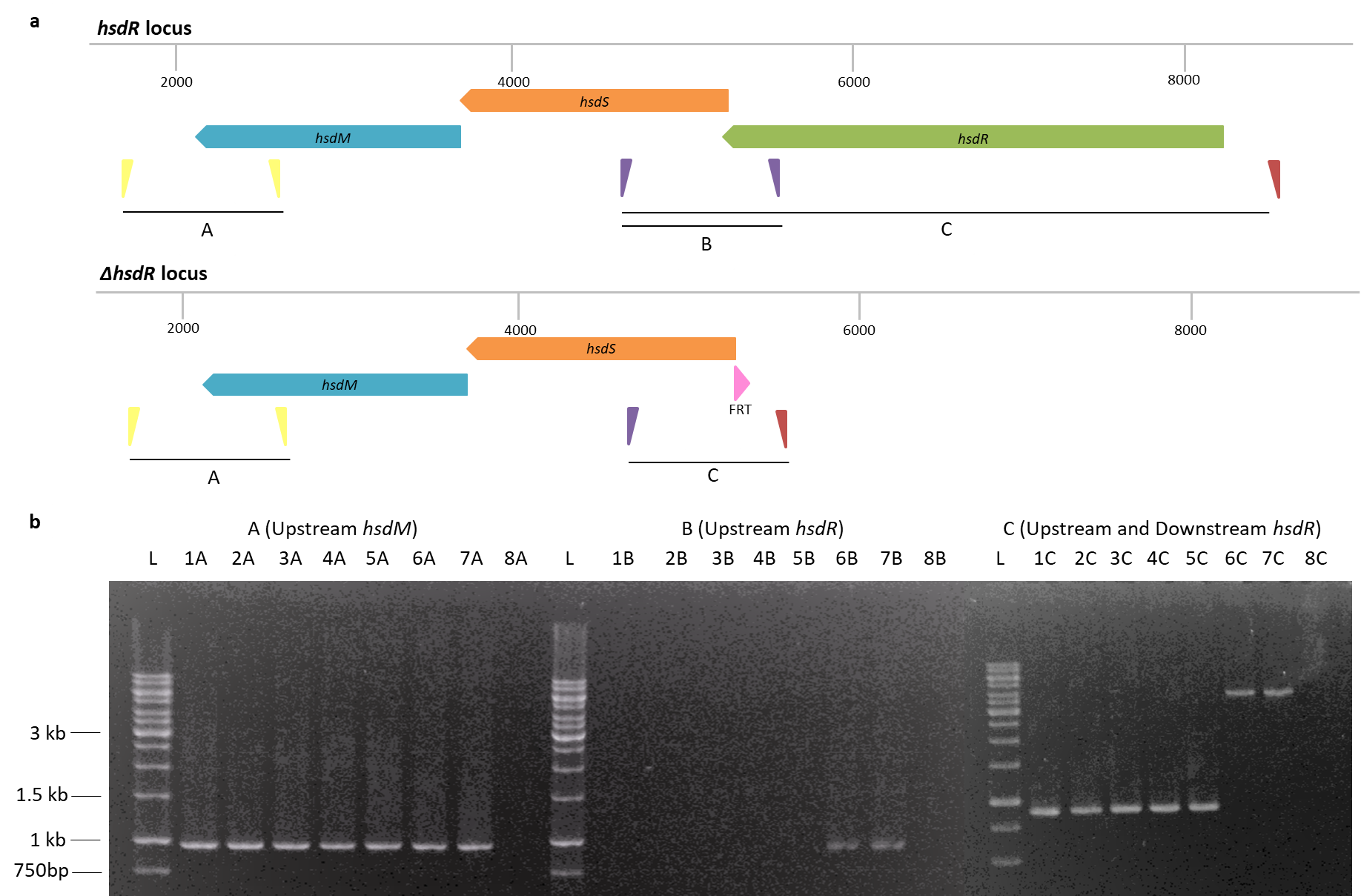
**

**Figure A. Verification of *hsdR* deletion in reduced *S. meliloti* strains by diagnostic PCR.** (a) Schematic of primer set locations (A, B and C) at the *hsdR* gene locus in wildtype *S. meliloti* when the *hsdR* gene is present (*hsdR* locus), and when the *hsdR* gene is replaced with FRT-Km/Nm-FRT cassette, which was subsequently excised via Flp recombinase (*∆hsdR* locus). The pink arrow indicates an FRT site following loss of the FRT-Km/Nm-FRT cassette. (b) Gel electrophoresis of diagnostic colony PCR conducted with primer sets A, B, and C on wildtype and designer *S. meliloti* strains. Expected band size for primer set A is 953 bp if the *hsdR* gene is present or absent. Expected band size for primer set B is 912 bp if the *hsdR* gene is present, and no band is expected if the *hsdR* gene is absent. Expected band size for primer set C is 4013 bp if the *hsdR* gene is present, and 787 bp if the *hsdR* gene is absent. L, 1 kb ladder. 1, RmP3909 ∆pSymAB ∆*hsdR*. 2, RmP3952 ∆pSymB Δ*hsdR*. 3, RmP3953 ∆pSymA Δ*hsdR*. 4, RmP3954 Δ*hsdR*. 5, Rm5000 ∆pSymA Δ*hsdR* Rif^R^. 6, RmP110 wildtype. 7, RmP110 wildtype isolated gDNA. 8, no DNA control.

**
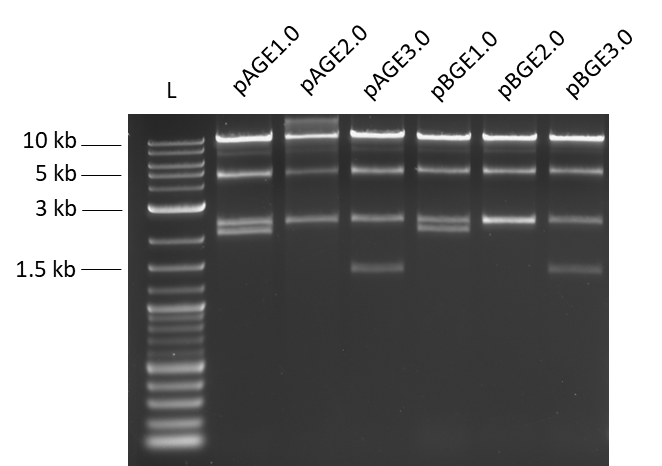
**

**Figure B. Diagnostic restriction digest of pAGE and pBGE vectors.** MHS vectors (pAGE1.0, pAGE2.0, pAGE3.0, pBGE1.0, pBGE2.0, pBGE3.0) were assembled in yeast, transformed into *E. coli* Epi300 cells, and induced to high copy with arabinose. Vector DNA was extracted and pAGE and pBGE vectors were digested with I-CeuI, I-SceI, PacI, and PmeI to confirm correct assembly. L, 2-log ladder.

**Figure C**. **Vector stability assay of pAGE2.0 in *S. meliloti* over 50 generations.** *S. meliloti* strain RmP4122 ∆pSymA Δ*hsdR* was transformed with pAGE2.0 by electroporation. Three independent cultures were grown in non-selective media (LBmc 38 μM FeCl_3_) for a total of approximately 50 generations. The cultures were plated on non-selective (LBmc 38 μM FeCl_3_) and selective (LBmc 38 μM FeCl_3_ Tc 5 μg mL^-1^) media every 10 generations and were subsequently subcultured into fresh non-selective media. The percentage of RmP4122 ∆pSymA Δ*hsdR* colonies that survived when plated on non-selective media but were unable to grow on selective media after each subculturing event was determined as an indication of vector stability


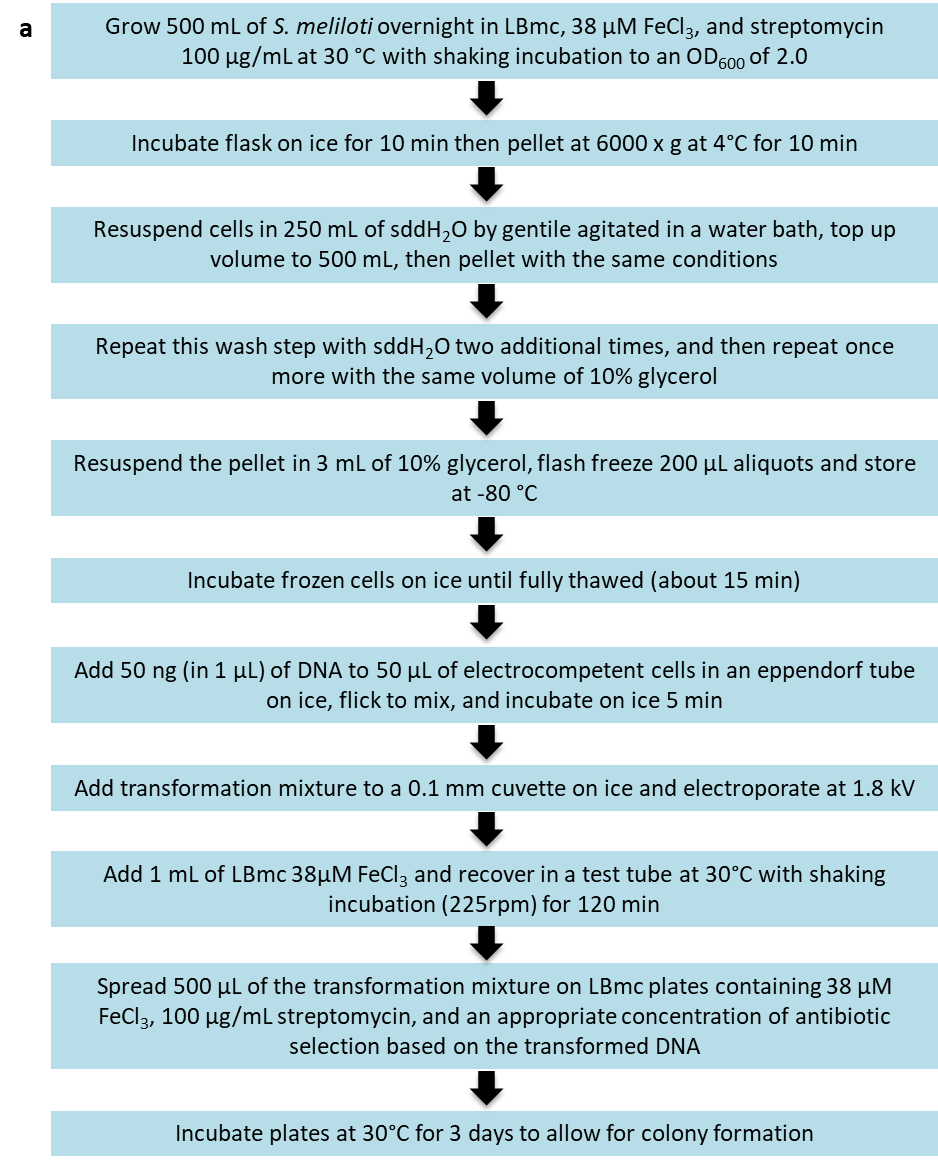


**Figure D.** **Workflow of optimized electroporation transformation protocol for *S. meliloti*.**


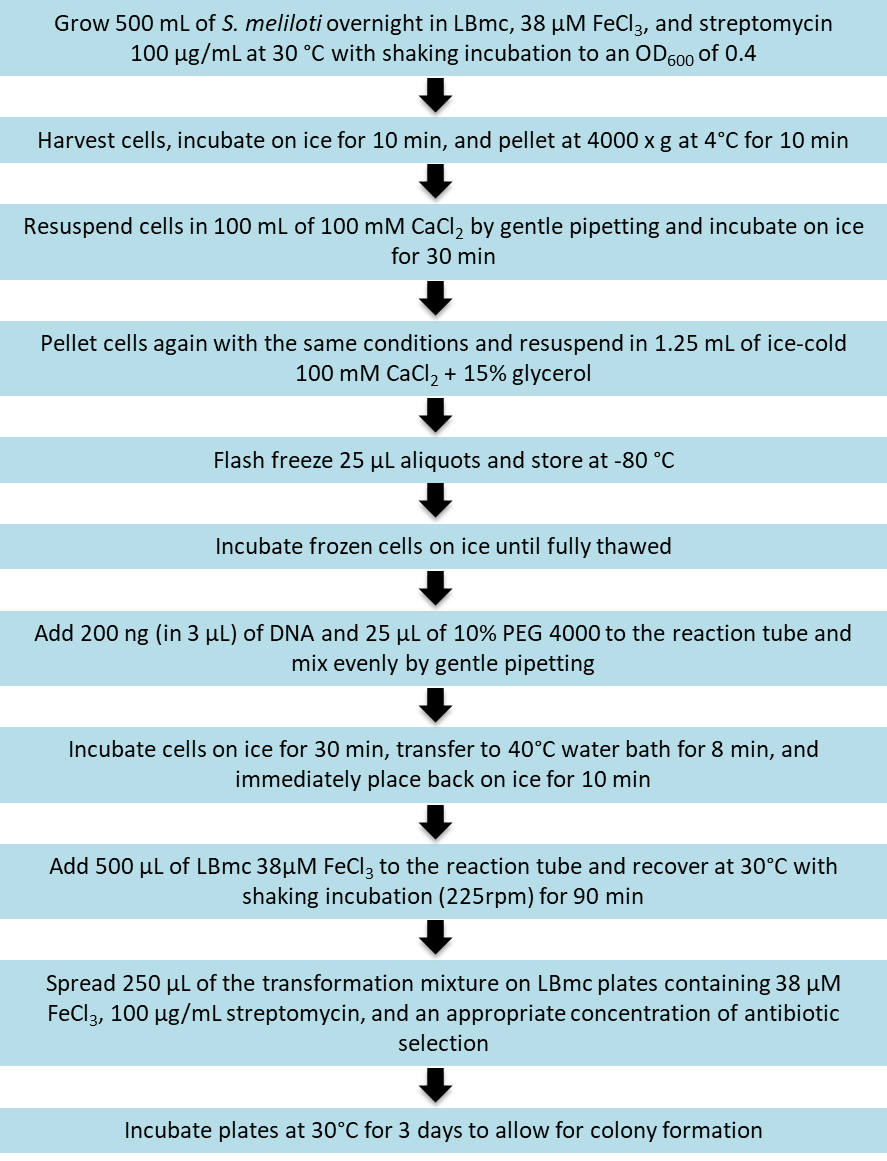


**Figure E.** **Workflow of optimized PEG-mediated transformation protocol for *S. meliloti*.**


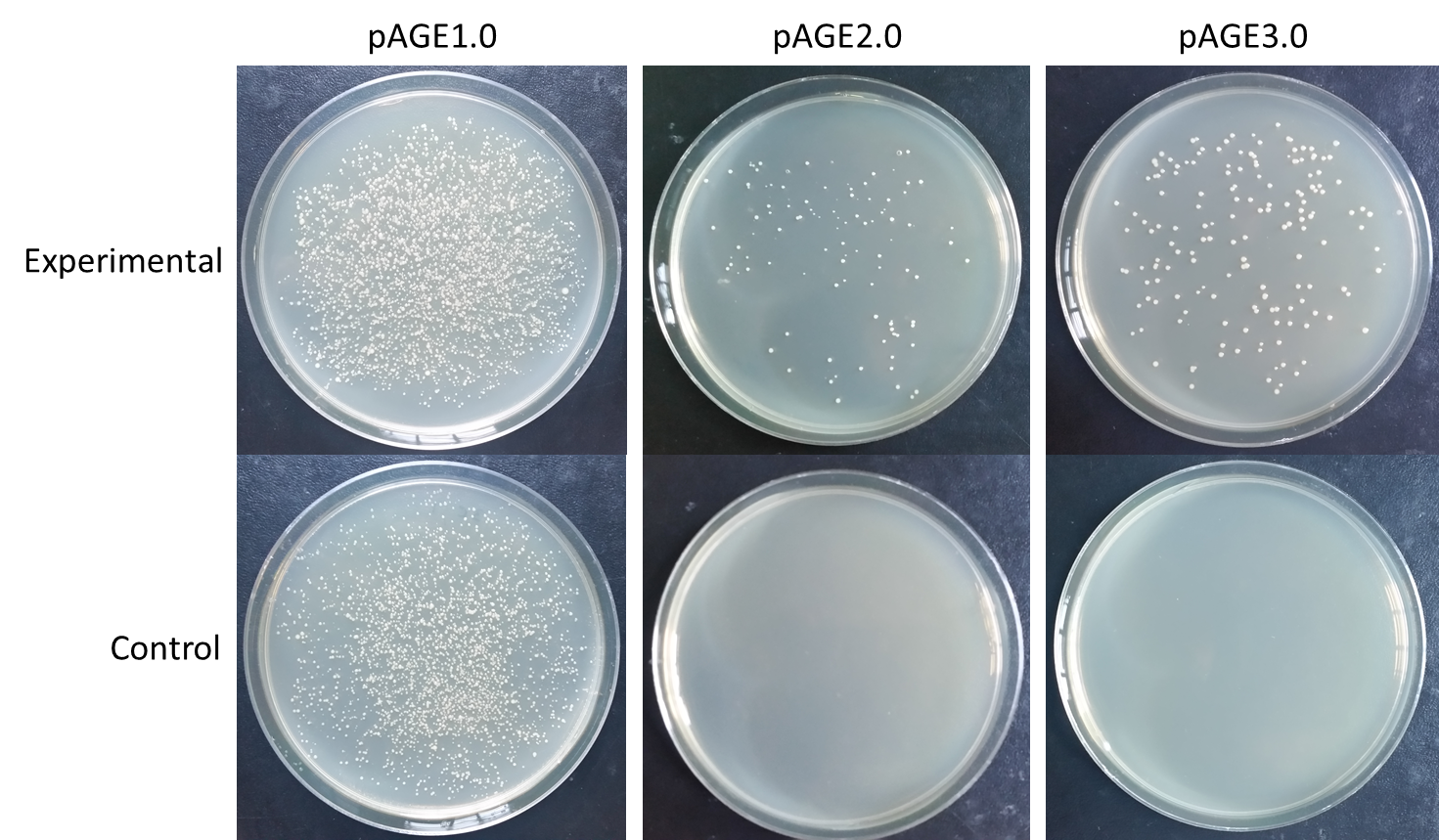


**Figure F. PEG-mediated transformation of pAGE vectors into *S. meliloti*.** Experimental and control plates from PEG-mediated transformation of pAGE1.0, pAGE2.0 and pAGE3.0 into *S. meliloti* RmP4122 ∆pSymA Δ*hsdR*. Control plates contain *S. meliloti* RmP4122 ∆pSymA Δ*hsdR* cells transformed with no DNA. pAGE1.0 transformation results were discarded due to the comparable number of colonies consistently observed on experimental and control plates.

**
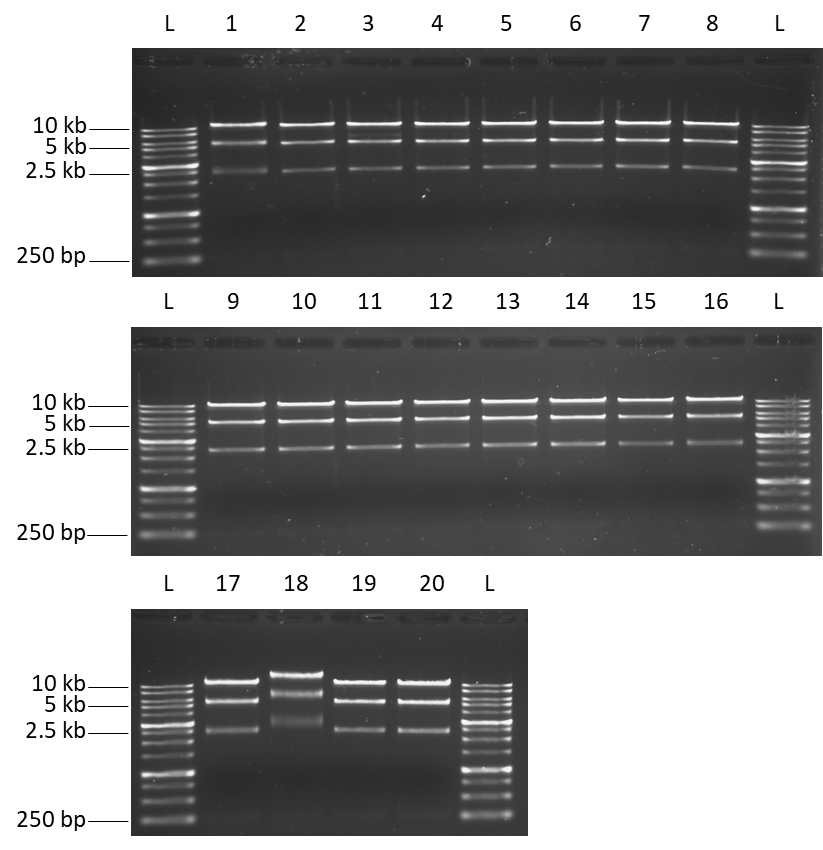
**

**Figure G**. **EcoRV-HF diagnostic digest of pAGE1.0 vectors extracted from 20 *E. coli* colonies following conjugation from *S. meliloti* to *E. coli*.** Expected band sizes of the pAGE1.0 vector following diagnostic restriction digest by EcoRV-HF are 10,288 bp, 5235 bp, 2377 bp, and 229 bp following gel electrophoresis on a 1% agarose gel. L, 1 kb ladder. 1-20, the 20 pAGE1.0 vectors extracted from *E. coli* and digested. Notes: 1) the 229 bp band is very faint and barely visible in this image; 2) clone number 3 (extra band between 10,288 bp and 5235 bp) and 18 showed incorrect digest pattern.

**
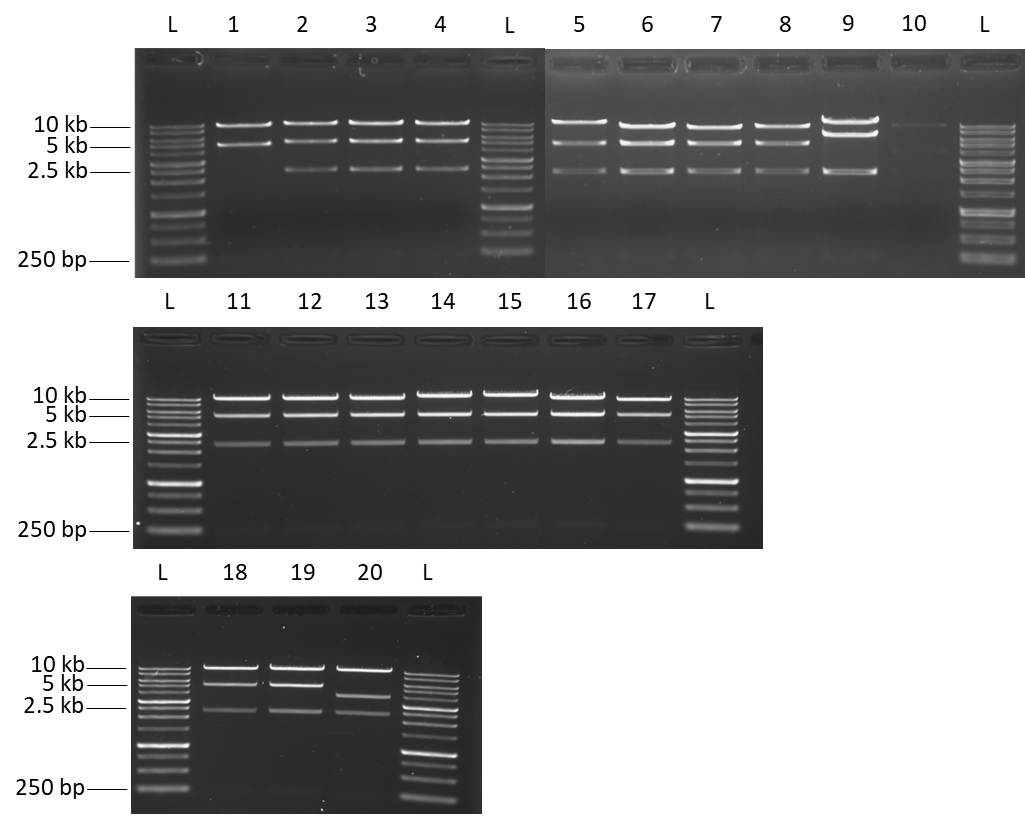
**

**Figure H**. **EcoRV-HF diagnostic digest of pAGE1.0 vectors extracted from 20 *E. coli* colonies following conjugation from *S. meliloti* to *P. tricornutum.*** Following conjugation, pAGE1.0 vectors were isolated from *P. tricornutum*, transformed into *E. coli*, induced to high copy and isolated again. Expected band sizes of the pAGE1.0 vector following diagnostic restriction digest by EcoRV-HF are 10,288 bp, 5235 bp, 2377 bp, and 229 bp following gel electrophoresis on a 1% agarose gel. L, 1 kb ladder. 1-20, the 20 pAGE1.0 vectors extracted from *E. coli* and digested. Notes: 1) the 229 bp band is very faint and barely visible in this image; 2) a total of 30 vectors were screened but only data for 20 is shown.

**
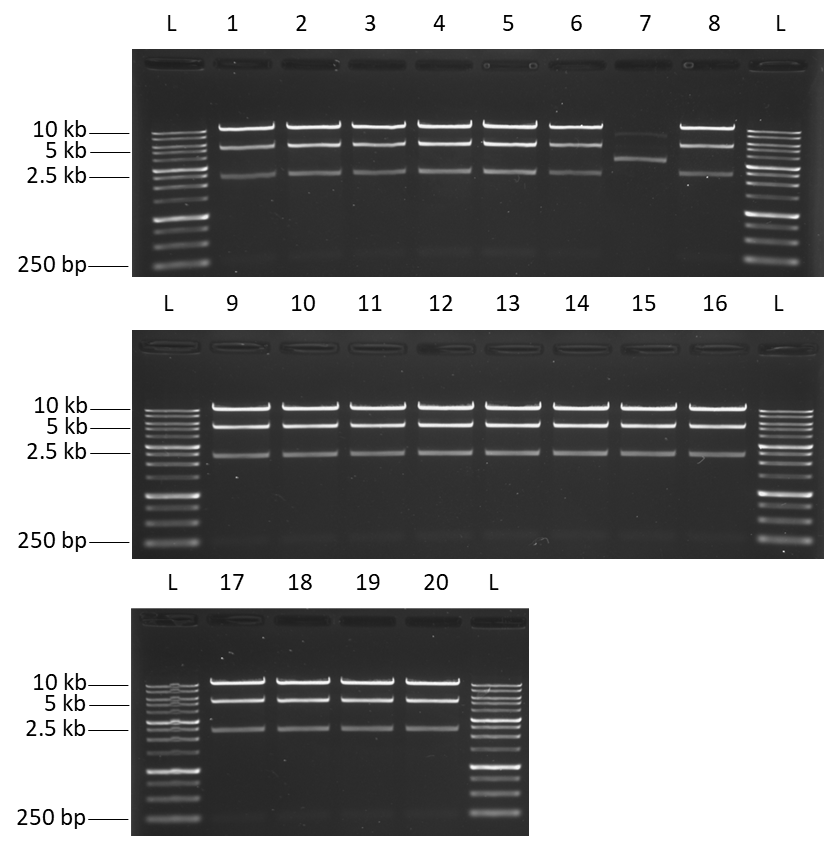
**

**Figure I**. **EcoRV-HF diagnostic digest of pAGE1.0 vectors extracted from 20 *E. coli* colonies following conjugation from *S. meliloti* to *S. cerevisiae.*** Following conjugation, pAGE1.0 vectors were isolated from *S. cerevisiae*, transformed into *E. coli*, induced to high copy and isolated again. Expected band sizes of the pAGE1.0 vector following diagnostic restriction digest by EcoRV-HF are 10,288 bp, 5235 bp, 2377 bp, and 229 bp following gel electrophoresis on a 1% agarose gel. L, 1 kb ladder. 1-20, the 20 pAGE1.0 vectors extracted from *E. coli* and digested. Notes: 1) The 229 bp band is very faint and barely visible in this image.
