# Supplementary material for: Designer Sinorhizobium meliloti strains and multi-functional vectors enable direct inter-kingdom DNA transfer
Source: PLoS One. 2019 Jun 17;14(6):e0206781. doi: 10.1371/journal.pone.0206781 (PMC6576745; doi:10.1371/journal.pone.0206781)
Supplement: S2 File — Table A. Vector stability of pAGE2.0 in S. meliloti RmP4122 ∆pSymA ΔhsdR. Table B. Efficiency of DNA transfer methods to S. meliloti RmP4122 ∆pSymA ΔhsdR including PEG-mediated transformation, electroporation and conjugation of pAGE vectors. Table C. Efficiency of pAGE1.0 conjugation from S. meliloti to recipient organisms and ratio of donor to recipient cells involved. Table D. Analysis of pAGE1.0 vectors recovered from conjugations from S. meliloti to E. coli, S. cerevisiae, and P. tricornutum. Table E. List of oligonucleotides used in this study. Table F. List of strains used in this study. Table G. List of vectors used in this study. (DOCX) [file pone.0206781.s002.docx]

**Designer *Sinorhizobium meliloti* strains and multi-functional vectors enable direct inter-kingdom DNA transfer**

Stephanie L. Brumwell^1^, Michael R. MacLeod^2^, Tony Huang^1^, Ryan R. Cochrane^1^, Rebecca S. Meaney^3^, Maryam Zamani^2^, Ola Matysiakiewicz^4^, Kaitlyn N. Dan^1^, Preetam Janakirama^3^, David R. Edgell^1^, Trevor C. Charles^4^, Turlough M. Finan^2^, Bogumil J. Karas^1,3*^

^1^Department of Biochemistry, Schulich School of Medicine and Dentistry, Western University, London, ON, Canada

^2^Department of Biology, McMaster University, Hamilton, ON, Canada

^3^Designer Microbes Inc., London, ON, Canada

^4^Department of Biology, University of Waterloo, Waterloo, ON, Canada

Corresponding author:

Bogumil J. Karas: bkaras@uwo.ca

**Table A.** **Vector stability of pAGE2.0 in *S. meliloti* RmP4122 ∆pSymA Δ*hsdR*.** *S. meliloti* strain RmP4122 ∆pSymA Δ*hsdR* was transformed with pAGE2.0 by electroporation. Three independent cultures were grown in non-selective media (LBmc 38 μM FeCl_3_) for a total of approximately 50 generations. The cultures were plated on non-selective (LBmc 38 μM FeCl_3_) and selective (LBmc 38 μM FeCl_3_ Tc 5 μg mL^-1^) media every 10 generations and were subsequently subcultured into fresh non-selective media. The number of colonies unable to grow on selective media (LBmc 38 μM FeCl_3_ Tc 5 μg mL^-1^) following subculturing in nonselective media (LBmc 38 μM FeCl_3_) was determined and averaged for the three independent cultures.

| **Number of Times Subcultured** | **Average Number of Doublings/Subculture** | **Number of colonies unable to grow on selective media (LBmc 38 μM FeCl_3_ Tc 5 μg mL^-1^)** | | | |
| --- | --- | --- | --- | --- | --- |
|  |  | **Sample 1** | **Sample 2** | **Sample 3** | **Sample Avg** |
| 0 | 0 | 3 | 2 | 2 | 2.3 |
| 1 | 9.65 | 7 | 4 | 6 | 5.6 |
| 2 | 9.5 | 11 | 5 | 16 | 10.6 |
| 3 | 9.53 | 13 | 15 | 19 | 15.6 |
| 4 | 9.8 | 20 | 16 | 19 | 18.3 |
| 5 | 9.85 | 24 | 24 | 28 | 25.3 |

**Table B.** **Efficiency of** **DNA transfer methods to *S. meliloti* RmP4122 ∆pSymA Δ*hsdR* including PEG-mediated transformation, electroporation and conjugation of pAGE vectors.** The pAGE plasmids were moved into *S. meliloti* RmP4122 ∆pSymA Δ*hsdR* through three methods including electroporation, PEG-mediated transformation, and conjugation from *E. coli* ECGE101 conjugative donor. Transformation efficiency for PEG-mediated transformation and electroporation is reported as CFU μg^-1^ of DNA, while conjugation efficiency is reported as transconjugants/recipient. Mean is the average of three biological and three technical replicates. *pAGE1.0 for the PEG-mediated transformation method consistently results in a similar number of colonies observed on the experimental and negative control plates (which can be seen in S1 Figure F).

| **Method** | **Vector** | **Colony Counts** | | | | **Transformation Efficiency (CFU/ug DNA)** | | | |
| --- | --- | --- | --- | --- | --- | --- | --- | --- | --- |
|  |  | **Exp 1** | **Exp 2** | **Exp 3** | **Mean** | **Exp 1** | **Exp 2** | **Exp 3** | **Mean** |
| **PEG-mediated**  **Transformation** | pAGE1.0* | n/a | n/a | n/a | n/a | n/a | n/a | n/a | n/a |
|  | pAGE2.0 | 65 | 94 | 95 | 85 | 1.31 x 10^3^ | 1.89 x 10^3^ | 1.90 x 10^3^ | 1.70 x 10^3^ |
|  | pAGE3.0 | 114 | 123 | 148 | 128 | 2.29 x 10^3^ | 2.47 x 10^3^ | 2.97 x 10^3^ | 2.58 x 10^3^ |
| **Electroporation** | pAGE1.0 | 144 | 107 | 42 | 97 | 2.88 x 10^4^ | 2.14 x 10^4^ | 8.40 x 10^3^ | 1.95 x 10^4^ |
|  | pAGE2.0 | 160 | 112 | 29 | 100 | 3.21 x 10^5^ | 2.24 x 10^5^ | 5.80 x 10^4^ | 2.01 x 10^5^ |
|  | pAGE3.0 | 126 | 126 | 41 | 97 | 2.52 x 10^5^ | 2.53 x 10^5^ | 8.20 x 10^4^ | 1.95 x 10^5^ |
| **Conjugation** |  | | | | | **Conjugation Efficiency (transconjugants/recipient)** | | | |
|  | pAGE1.0 | 93 | 83 | 83 | 86 | 0.490 | 0.298 | 0.453 | 0.414 |
|  | pAGE2.0 | n/a | n/a | n/a | n/a | n/a | n/a | n/a | n/a |
|  | pAGE3.0 | n/a | n/a | n/a | n/a | n/a | n/a | n/a | n/a |

**Table C.** **Efficiency of pAGE1.0 conjugation from *S. meliloti* to recipient organisms and ratio of donor to recipient cells involved.** Average conjugation efficiency for each donor-recipient pair, as determined by three biological replicates and three technical replicates for each pair. Post-conjugation non-selective plates and pAGE1.0 selective plates were used to determine the conjugation efficiency (calculated as transconjugants/recipient) between each donor and recipient pair. Pre-conjugation plates selecting for the conjugative participants and colony counts were used to determine the ratio of donor to recipient organism going into the conjugation mixture.

| **Donor** | **Recipient** | **Ratio (donor:recipient)** | **Conjugation Efficiency**  **(transconjugants/recipient)** |
| --- | --- | --- | --- |
| *S. meliloti* | *E. coli* | 2:1 | 2.2 x 10^-1^ |
| *S. meliloti* | *P. tricornutum* | 74:1 | 9.4 x 10^-5^ |
| *S. meliloti* | *S. cerevisiae* | 26:1 | 8.0 x 10^-6^ |

**Table D. Analysis of pAGE1.0 vectors recovered from conjugations from *S. meliloti* to *E. coli, S. cerevisiae,* and *P. tricornutum***. Following conjugation, sixty pAGE1.0 vectors from *S. cerevisiae* and *P. tricornutum* transconjugants were isolated and transformed into *E. coli.* The number of transformations resulting in *E. coli* colonies, out of 60, and the average number of *E. coli* colonies was determined. Then, 20 of these isolated vectors, as well as those isolated from *E. coli* transconjugants were induced with arabinose and once again isolated to obtain high quality DNA. The number of correct vectors were determined by diagnostic restriction digests of the pAGE1.0 vectors with EcoRV-HF.

| **Recipient** | **Number of vectors rescued in *E. coli*** | **Average number of *E. coli* colonies** | **Number of correct vectors digested with EcoRV** | **Liklihood of obtaining correct vectors** |
| --- | --- | --- | --- | --- |
| *E. coli* | n/a | n/a | 18/20 | 90% |
| *P. tricornutum* | 16/60 (<25 *E. coli* colonies) | 5 | 0/10 | 56% |
|  | 42/60 (>25 *E. coli* colonies) | 316 | 16/20 |  |
| *S. cerevisiae* | 59/60 | 15 | 19/20 | 93% |

**Table E.** **List of oligonucleotides used in this study.**

| **Name** | **Sequence (5’ to 3’)** | **Description** |
| --- | --- | --- |
| **Development of Δ*hsdR* strains** | | |
| Km_cas_F | CGACGTCTCGGTCGCCGAAGAGACCTTTGCCGAGGCTGCGGAATAGCCCGTGTAGGCTGGAGCTGCTTC | Forward Km cassette primer to delete *hsdR* |
| Km_cas_R | CTCAACCTGCCGTCTCCAGTGAAGGTGGCGGGGAGACTGGAAGCCAAAGTATTCCGGGGATCCGTCGACC | Reverse Km cassette primer to delete *hsdR* |
| Development of ECGE101 | | |
| DAP1 | ATGTTCACGGGAAGTATTGTC | Forward *dapA* region from βDH10B |
| DAP2 | CAGCAAACCGGCATGCTTAA | Reverse *dapA* region from βDH10B |
| **Diagnostic PCR of Δ*hsdR* strains** | | |
| hsdR_U_F | ggtacccggggatcctctagGACATAGCATCCGTATCAGTTGGG | Forward primer for positive control in diagnostic colony PCR to validate deletion of *hsdR* |
| hsdR_U_R | ggtcaggcttccggcattatCATCCCGCCGTCATCGTCG | Reverse primer for positive control in diagnostic colony PCR to validate deletion of *hsdR* |
| hsdM_U_F | ggtacccggggatcctctagCAAAGGACGGCACTTATTCAACGG | Forward primer for diagnostic colony PCR to validate deletion of *hsdR* |
| hsdM_R_R | ggtcaggcttccggcattatAGCCGGACGATGGACGAGG | Reverse primer for diagnostic colony PCR to validate deletion of *hsdR* |
| hsdR_D_R | gcctgcaggtcgactctagGATCAAGCTACTCGATCAGGTGC | Reverse primer for diagnostic colony PCR to validate deletion of *hsdR* |
| **Assembly of MHS vectors** | | |
| D117_F | GTCCTTTTACAGCCAGTAGTGCTCGCCGCAGTCGAGCGACAGGGCGAAGCCCGTTTAAACccggggatccggtgattgat | Forward Sp/Sm resistance cassette (pAGE1.0, pBGE1.0) |
| D117_R | TACCGAAAAAATCGCTATAATGACCCCGAAGCAGGGTTATGCAGCGGAAGATTTAATTAAggatccggtgattgattgag | Reverse Sp/Sm resistance cassette (pAGE1.0, pBGE1.0) |
| D118_F | tttcacaaaacggtttacaagcataaagcttgctcaatcaatcaccggatccTTAATTAAATCTTCCGCTGCATAACCCT | Forward oriT (all pAGE and pBGE) |
| D118_R | CCTAAAAGACATAGCACGCGACAAGCACGAGCGAGATATCCCAATCAAGCTAGTATCGATGATCGTCTTGCCTTGCTCGT | Reverse oriT (all pAGE and pBGE) |
| D119_F | AGAAGAGGCACTTCGAGCTGTAAGTACATCACCGACGAGCAAGGCAAGACGATCATCGATACTAGCTTGATTGGGATATC | Forward Ntc resistance cassette (all pAGE and pBGE) |
| D119_R | GTCGCGATCGTGCGACGCCACGAACGGAGTGTTCGCTACCTTAGGACCGTTATAGTTACGGACGTTTTCACTCTCGAGCA | Reverse Ntc resistance cassette (all pAGE and pBGE) |
| D120_F | TAAAAAACCTGTGCTCGAGAGTGAAAACGTCCGTAACTATAACGGTCCTAAGGTAGCGAACACTCCGTTCGTGGCGTCGC | Forward *repA2B2C2* (all pAGE) |
| D120_R | CCAGCCCAGCGGCGAGGGCAACCAGCTCGACTATATTACCCTGTTATCCCTAGCGTAACTGAACCATCCGGTGCAGTCGT | Reverse *repA2B2C2* (all pAGE) |
| D121_F | TGACCGAGCAACGACTGCACCGGATGGTTCAGTTACGCTAGGGATAACAGGGTAATATAGTCGAGCTGGTTGCCCTCGCC | Forward pCC1BAC-yeast part 1 (all pAGE and pBGE) |
| D121_R | TTCAACCGGTTGAGTATTGAGCGTATGTTTTGGAATAACAGGCGCACGCTTCATTATCTAATCTCCCAGCGTGGTTTAAT | Reverse pCC1BAC-yeast part 1 (all pAGE and pBGE) |
| D122_F | ATTAAACCACGCTGGGAGATTAGATAATGAAGCGTGCGCCTGTTATTCCAAAACATACGCTCAATACTCAACCGGTTGAA | Forward pCC1BAC-yeast part 2 (all pAGE and pBGE) |
| D122_R | acaaaacggtttacaagcataaagcttgctcaatcaatcaccggatccccggGTTTAAACGGGCTTCGCCCTGTCGCTCG | Reverse pCC1BAC-yeast part 2 (all pAGE and pBGE) |
| D200_F | AGCCAGTAGTGCTCGCCGCAGTCGAGCGACAGGGCGAAGCCCGTTTAAACgagcgccagaaggccgccagagaggccgag | Forward Tet gene from pRK7813 (pAGE2.0, pBGE2.0) |
| D200_R | ATCGCTATAATGACCCCGAAGCAGGGTTATGCAGCGGAAGATTTAATTAAgatcagacgctgagtgcgcttcaaatcatc | Reverse Tet gene from pRK7813 (pAGE2.0, pBGE2.0) |
| D201_F | AGCCAGTAGTGCTCGCCGCAGTCGAGCGACAGGGCGAAGCCCGTTTAAACggggtgggcgaagaactccagcatgagatc | Forward NptII gene from pKNT253 (pAGE3.0, pBGE3.0) |
| D201_R | ATCGCTATAATGACCCCGAAGCAGGGTTATGCAGCGGAAGATTTAATTAAagcttcacgctgccgcaagcactcagggcg | Reverse NptII gene from pKNT253 (pAGE3.0, pBGE3.0) |
| D234_F | GTGCTCGAGAGTGAAAACGTCCGTAACTATAACGGTCCTAAGGTAGCGAAGCCTTATCCTTCTCTTATCCGACCTGCGGG | Forward *repA1B1C1* (all pBGE) |
| D234_R | GGCGAGGGCAACCAGCTCGACTATATTACCCTGTTATCCCTAGCGTAACTGGGGCAAGATCGTATTGTTGACAGCTGTCA | Reverse r*epA1B1C1* (all pBGE) |

**Table F. List of strains used in this study.**

| **Strain** | **Description** | **Resistance** | **Reference or Source** |
| --- | --- | --- | --- |
| ***Sinorhizobium meliloti*** | | | |
| Rm5000 | SU47 *rif-5* | Rif | [1] |
| RmP110 | RmP1021, wild type SU47 *str-21* with wild-type *pstC* allele | Sm | [2] |
| RmP3491 | Rm5000 (pTE3::*rctB* of *R. etli*) | Rif Tc | [3] |
| RmP3500 | ΔpSymA ΔpSymB with *engA-tRNAarg-rmlC* moved to chromosome | Sm | [3] |
| RmP3901 | RmP3500 with upstream and downstream regions of NGR234 *bacA* gene at *bacA* loci | Sm Gm | [4] |
| RmP3909 | ΔpSymA ΔpSymB with *engA-tRNAarg-rmlC* moved to chromosome; *bacA* gene replaced with *bacA* from Rm2011; derived from RmP3500 | Sm | [4] |
| RmP3910 | RmP3901 with *bacA* gene replaced with NGR234 *bacA* | Sm | [4] |
| RmP3950 | RmP3910 with pSymB from RmP3491 | Sm | [4] |
| RmP3952 | RmP3909 pSymA+ ΔpSymB | Sm | [4] |
| Rmp3953 | RmP3909 ΔpSymA pSymB+ | Sm | [4] |
| RmP3954 | RmP3909 pSymA+ pSymB+ | Sm | [4] |
| RmP3975 | RmP110 (pTH3144) Δ*hsdR hsdR* gene replaced with Km/Nm cassette | Sm Nm | Zamani, unpublished. |
| RmP4098 | RmP3950 ΔpSymA *hsdR*::Nm | Sm Nm | Zamani, unpublished. |
| RmP4122 | RmP4098 ΔpSymA *hsdR*::FRT | Sm Nm^S^ | This study. |
| RmP4124 | RmP3953 Δ*hsdR hsdR*::FRT | Sm Nm^S^ | This study. |
| RmP4125 | RmP3954 Δ*hsdR hsdR*::FRT | Sm Nm^S^ | This study. |
| RmP4246 | Rm5000 ΔpSymA | Rif | Bindra, Sather and Finan, unpublished. |
| RmP4258 | RmP3909 Δ*hsdR hsdR*::FRT | Sm Nm^S^ | This study. |
| RmP4260 | RmP3952 Δ*hsdR hsdR*::FRT | Sm Nm^S^ | This study. |
| RmP4262 | RmP4246 Δ*hsdR hsdR*::FRT | Rif Nm^S^ | This study. |
| ***Escherichia coli*** | | | |
| DH5α | F- *endA1 hsdR17 supE44 thi-1 λ- recA1 gyrA96 relA1 Φ80dlacZΔM15* |  | [5] |
| MT616 | MT607 (pRK600); contains RK2 transfer genes | Cm | [6] |
| M1449 | DH5α (pTH2505) | Tc | [7] |
| βDH10B | ∆*dapA* |  | [8] |
| M2453 | DH5α (pTH3142; *hsdR* upstream and downstream regions cloned in pUCP30T)) | Gm | Zamani, unpublished |
| M2459 | M2456 (Km cassette introduced to delete *hsdR*); pKD46 | Gm Km Amp | Zamani, unpublished |
| ECGE101 | Epi300 with the *dapA* gene deleted (∆*dapA)* |  | This study. |

**Table G.** **List of vectors used in this study.**

| **Vector** | **Description** | **Resistance** | **Reference or Source** |
| --- | --- | --- | --- |
| pUCP30T | ColE1 cloning vector in *S. meliloti,* oriT | Gm | [9] |
| pKD46 | Expressed *lambda red recombinase* genes | Amp | [10] |
| pTH2505 | Expresses *flp* *recombinase* under control of PCA-inducible promoter | Tc | [7] |
| pTA-Mob | Broad-host-range mobilization plasmid | Gm | [11] |
| pAGE1.0 | MHS vector (as described in the main text) | Sp (*S. meliloti*)  Cm (*E. coli*)  HIS3 (*S. cerevisiae*)  Ntc (*P. tricornutum)* | This study |
| pAGE2.0 | MHS vector (as described in the main text) | Sp (*S. meliloti*)  Cm (*E. coli*)  HIS3 (*S. cerevisiae*)  Ntc (*P. tricornutum)* | This study |
| pAGE3.0 | MHS vector (as described in the main text) | Sp (*S. meliloti*)  Cm (*E. coli*)  HIS3 (*S. cerevisiae*)  Ntc (*P. tricornutum)* | This study |
| pBGE1.0 | MHS vector (as described in the main text) | Sp (*S. meliloti*)  Cm (*E. coli*)  HIS3 (*S. cerevisiae*)  Ntc (*P. tricornutum)* | This study |
| pBGE2.0 | MHS vector (as described in the main text) | Sp (*S. meliloti*)  Cm (*E. coli*)  HIS3 (*S. cerevisiae*)  Ntc (*P. tricornutum)* | This study |
| pBGE3.0 | MHS vector (as described in the main text) | Sp (*S. meliloti*)  Cm (*E. coli*)  HIS3 (*S. cerevisiae*)  Ntc (*P. tricornutum)* | This study |

**References**

1. Finan TM, Hartweig E, LeMieux K, Bergman K, Walker GC, Signer ER. General transduction in Rhizobium meliloti. J Bacteriol. 1984;

2. Yuan ZC, Zaheer R, Finan TM. Regulation and properties of PstSCAB, a high-affinity, high-velocity phosphate transport system of Sinorhizobium meliloti. J Bacteriol. 2006; doi:10.1128/JB.188.3.1089-1102.2006

3. diCenzo GC, Zamani M, Milunovic B, Finan TM. Genomic resources for identification of the minimal N2-fixing symbiotic genome. Environ Microbiol. 2016;18: 2534–2547. doi:10.1111/1462-2920.13221

4. diCenzo GC, Zamani M, Ludwig HN, Finan TM. Heterologous Complementation Reveals a Specialized Activity for BacA in the *Medicago* – *Sinorhizobium meliloti* Symbiosis. Mol Plant-Microbe Interact. 2017; doi:10.1094/MPMI-02-17-0030-R

5. Hanahan D. Studies on transformation of Escherichia coli with plasmids. J Mol Biol. 1983; doi:10.1016/S0022-2836(83)80284-8

6. Finan TM, Kunkel B, De Vos GF, Signer ER. Second symbiotic megaplasmid in Rhizobium meliloti carrying exopolysaccharide and thiamine synthesis genes. J Bacteriol. 1986; doi:10.1128/jb.167.1.66-72.1986

7. White CE, Gavina JMA, Morton R, Britz-Mckibbin P, Finan TM. Control of hydroxyproline catabolism in Sinorhizobium meliloti. Mol Microbiol. 2012;85: 1133–1147. doi:10.1111/j.1365-2958.2012.08164.x

8. Rowe-magnus DA. Integrase-directed recovery of functional genes from genomic libraries. Nucleic Acids Res. 2009; doi:10.1093/nar/gkp561

9. Schweizer, H.P., Klassen, T.R., Hoang T. Improved methods for gene analysis and expression in Pseudomonas. Mol Biol Pseudomonads Am Soc Microbiol. 1996; 229–237.

10. Smith GR. Homologous recombination in procaryotes. Microbiol Rev. 1988; doi:10.1111/j.1365-2958.2004.04197.x

11. Strand TA, Lale R, Degnes KF, Lando M, Valla S. A new and improved host-independent plasmid system for RK2-based conjugal transfer. PLoS One. 2014;9: 1–6. doi:10.1371/journal.pone.0090372
