# Supplementary material for: Correction: Designer Sinorhizobium meliloti strains and multi-functional vectors enable direct inter-kingdom DNA transfer
Source: PLoS One. 2019 Jul 9;14(7):e0219562. doi: 10.1371/journal.pone.0219562 (PMC6615600; doi:10.1371/journal.pone.0219562)
Supplement: S1 File — (PDF) [file pone.0219562.s001.pdf]

PLOS | ONE

RESEARCH ARTICLE

Designer *Sinorhizobium meliloti* strains and multi-functional vectors enable

Stephanie L. Brumwell[1], Michael R. MacLeod[2], Tony Huang[1], Ryan R. Cochrane[1], Rebecca S. Meaney[3], Maryam Zamani[2], Ola Matysiakiewicz[4], Kaitlyn N. Dan[1], Preetam Janakirama[3], David R. Edgell[1], Trevor C. Charles[4], Turlough M. Finan[2], Bogumil J. Karas[1,3]**

1 Department of Biochemistry, Schulich School of Medicine and Dentistry, Western University, London, ON, Canada, **2** Department of Biology, McMaster University, Hamilton, ON, Canada, **3** Designer Microbes Inc., London, ON, Canada, **4** Department of Biology, University of Waterloo, Waterloo, ON, Canada

* bkaras@uwo.ca

Check for updates

Abstract

Storage, manipulation and delivery of DNA fragments is crucial for synthetic biology applications, subsequently allowing organisms of interest to be engineered with genes or pathways to produce desirable phenotypes such as disease or drought resistance in plants, or for synthesis of a specific chemical product. However, DNA with high G+C content can be unstable in many host organisms including *Saccharomyces cerevisiae*. Here, we report the development of *Sinorhizobium meliloti*, a nitrogen-fixing plant symbioticα-Proteobacterium, as a novel host that can store DNA, and mobilize DNA to *E. coli*, *S. cerevisiae*, and the eukaryotic microalgae *Phaeodactylum tricornutum*. To achieve this, we deleted the *hsdR* restriction-system in multiple reduced genome strains of *S. meliloti* that enable DNA transformation with up to 1.4 x 10^5 and 2.1 x 10^3 CFU μg^{-1} of DNA efficiency using electroporation and a newly developed polyethylene glycol transformation method, respectively. Multi-host and multi-functional shuttle vectors (MHS) were constructed and stably propagated in *S. meliloti*, *E. coli*, *S. cerevisiae*, and *P. tricornutum*. We also developed protocols and demonstrated direct transfer of these MHS vectors via conjugation from *S. meliloti* to *E. coli*, *S. cerevisiae*, and *P. tricornutum*. The development of *S. meliloti* as a new host for inter-kingdom DNA transfer will be invaluable for synthetic biology research and applications, including the installation and study of genes and biosynthetic pathways into organisms of interest in industry and agriculture.

OPEN ACCESS

Citation: Brumwell SL, MacLeod MR, Huang T, Cochrane RR, Meaney RS, Zamani M, et al. (2019) Designer *Sinorhizobium meliloti* strains and multi-functional vectors enable. PLoS ONE 14(6): e0206781. https://doi.org/10.1371/journal.pone.0206781

Editor: Francisco Martinez-Abarca, Estacion Experimental del Zaidin - CSIC, SPAIN

Received: October 17, 2018

Accepted: May 31, 2019

Published: June 17, 2019

Copyright: © 2019 Brumwell et al. This is an open access article distributed under the terms of the Creative Commons Attribution License, which permits unrestricted use, distribution, and reproduction in any medium, provided the original author and source are credited.

Data Availability Statement: All relevant data are within the manuscript and its Supporting Information files.

Funding: This work was supported by a Genome Canada Grant (OGI-111) to T.C.C., T.M.F. and B.J.K. Research in the labs of D.R.E., T.C.C., T.M.F. and B.J.K. is also supported by Natural Sciences and Engineering Research Council of Canada (NSERC), RGPIN-2015-04800, RGPIN-2018-04754, RGPIN-2018-06481, and RGPIN-2018-06172. In addition, this study was supported by Designer Microbes

Introduction

The field of synthetic biology aims to utilize existing or novel biological parts and systems to create organisms that can help address global problems including increased demand for food, fuel, therapeutics, and high value chemicals. However, one of the major obstacles in synthetic biology is that many organisms of interest lack genetic tools such as autonomously replicating plasmids, well characterized promoters and terminators, selective markers, genome-editing

Inc. (DMI). The funder provided support in the form of salaries for R.S.M., and P.J., and the specific role of these authors are articulated in the 'Author Contributions' section.

Competing interests: B.J.K. is Chief Executive Officer of Designer Microbes Inc. and holds Designer Microbes Inc. stock. P.J. also holds Designer Microbes Inc. stock. The funder provided support in the form of salaries for R.S.M., and P.J. This competing interest does not alter our adherence to PLOS ONE policies on sharing data and materials.

tools, and protocols to uptake and install DNA [1,2]. This problem can be addressed by cloning whole chromosomes or large DNA fragments from an organism of interest in a surrogate host, where genetic tools are in place and manipulations can be performed [3–5]. Currently, the most common host for the capture and manipulation of DNA fragments is *Saccharomyces cerevisiae* [6–10], however returning cloned or engineered fragments to destination cells is still challenging due to the lack of direct transfer methods from *S. cerevisiae*, such as bacterial conjugation. In addition, *S. cerevisiae* cannot maintain large DNA fragments with G+C content >40% without additional engineering [11–13], and many industrially useful bacterial strains have a G+C content above this range. For example, *Streptomyces* species have a G+C content >65% and are important for the production of antibiotics (gentamicin, kanamycin, tetracycline, etc.) [14]. Therefore, it is desirable to develop a host to clone, maintain, manipulate, and transfer DNA fragments, including high G+C content, to bacterial and eukaryotic destination cells.

Sinorhizobium meliloti is an attractive host candidate for this application. *S. meliloti* is a Gram-negative α-Proteobacterium that forms a symbiotic relationship with legume plants where it fixes nitrogen in root nodules, and therefore is highly important in agriculture. *S. meliloti* model strain Rm1021 has a multipartite genome including a chromosome (3.65 Mb), pSymA megaplasmid (1.35 Mb), and pSymB chromid (1.68 Mb) [15]. Multiple derivatives of *S. meliloti* now exist that vary in genome size, nutrient requirements and generation time [16]. Recently, a derivative of *S. meliloti* with a minimal genome lacking the pSymA and pSymB replicons was developed, resulting in a 45% reduction of the genome [16]. Two essential genes were identified in the pSymB chromid, *engA* and $tRNA^{arg}$, and these genes were transferred to the main chromosome [17]. The replication origins of pSymA and pSymB were identified and characterized [18], and can be used to generate designer replicating plasmids. Additionally, several genetic tools have been developed for this species including vectors based on *repABC* origins taken from various α-Proteobacteria that can be propagated in *S. meliloti* [19]. Three of these vectors can be maintained within wildtype *S. meliloti* at one time, along with the endogenous pSymA and pSymB replicons. Utilizing these vectors, an *in vivo* cloning method with Cre/lox-mediated translocation of large DNA fragments to the *repABC*-based vector was established [19]. Therefore, *S. meliloti* is an attractive host organism due to its high G+C content genome (62%) [20], available origins of replication that could be used to maintain large DNA fragments, expanding genetic toolbox, and applications in agriculture.

S. meliloti is also an attractive host bacterium because it is able to move DNA to other organisms using several methods. Previous studies have demonstrated DNA transfer methods from *S. meliloti* through introduction of a Ti plasmid to plant species through mobilization of TDNA [21]. In addition, *S. meliloti* is able to move DNA to bacteria via conjugation in triparental mating experiments [19].

Installing a conjugative plasmid into *S. meliloti*, such as pTA-Mob, will allow for faster and more efficient transfer of DNA to a recipient organism. The conjugative pTA-Mob plasmid, which contains all of the genes required for direct DNA transfer via conjugation, can mobilize any additional cargo plasmid containing an origin of transfer (*oriT*). Conjugation is a particularly attractive method of DNA transfer to streamline the workflow for genetic manipulation of organisms. While transfer of DNA from *S. meliloti* to bacterial cells has been demonstrated, DNA transfer to eukaryotic cells, such as yeast and algae, has not been previously described.

Here, we utilized the reduced genome strains of *S. meliloti* [16] to create designer strains with the restriction-system removed and the conjugative pTA-Mob [22] plasmid installed. In addition, we used identified origins from *S. meliloti* pSymA and pSymB replicons to create multi-host shuttle (MHS) vectors that replicate in *S. meliloti*, *Escherichia coli*, *S. cerevisiae*, and *Phaeodactylum tricornutum*. The MHS vectors were tested for their ability to provide

antibiotic resistance and propagate in *S. meliloti*. These vectors were moved into *S. meliloti* strains via an optimized electroporation protocol, via a newly developed polyethylene glycol (PEG) method, or via conjugation from *E. coli*. Most notably, we have developed protocols to directly transfer the MHS vectors via conjugation from *S. meliloti* to *E. coli*, *S. cerevisiae*, and *P. tricornutum*.

Results and discussion

Development of designer bacterial strains

Initially, we engineered *S. meliloti* strains to remove the restriction-system (Δ*hsdR*) to allow for development of more efficient transformation methods. Disruption or deletion of the *hsdR* gene has been previously reported in the Rm1021 strain of *S. meliloti*, and deletion mutants were shown to have enhanced transformation efficiencies [19,23]. In our reduced *S. meliloti* strains, either lacking one or both of the pSymA or pSymB replicons, the *hsdR* gene was replaced by a FRT-Km/Nm-FRT cassette and the resulting *ΔhsdR*::Nm mutant allele was transferred to various background strains via transduction of Nm^r. The FRT-Km/Nm-FRT cassette was then removed by introducing an unstable plasmid (pTH2505) carrying the Flp recombinase, followed by curing of this plasmid. The *hsdR* deletion strains were verified to have the *hsdR* gene successfully deleted using diagnostic PCR with primers located upstream and downstream of the *hsdR* gene locus (Figure A in S1 File). *S. meliloti* RmP4098 ΔpSymA *ΔhsdR*, retaining the Km/Nm cassette and the pSymB chromid, was chosen as our host strain as it had the fastest doubling time when compared to the other reduced-genome strains [16]. *S. meliloti* *Δ*pSymA *ΔhsdR*, was transformed with the pTA-Mob plasmid [22] and used as the conjugative donor strain for all subsequent experiments (Fig 1A). In addition, reduced *S. meliloti* strains RmP3952 ΔpSymB Δ*hsdR* and RmP3909 ΔpSymAB Δ*hsdR* were developed using the same method described above.

Additionally, a designer *E. coli* strain was developed to simplify the current method of conjugation from *E. coli*. We used lambda red recombination to create an auxotrophic strain of *E. coli* Epi300, named ECGE101, by deleting the *dapA* gene. This gene is required for synthesis of diaminopimelic acid (DAP) [24], therefore ECGE101 requires DAP supplementation in the growth media and provides a useful, antibiotic-free method for counter-selecting *E. coli* after conjugation to *S. meliloti* or any other organism.

Design, assembly and characterization of multi-host shuttle vectors

With the reduced, restriction-system minus strains of *S. meliloti* created, and the origins of replications of the large megaplasmid and chromid (*repA1B1C1* and *repA2B2C2*) identified, MHS vectors were developed as cargo plasmids for conjugation from *S. meliloti* to various recipients. Six MHS vectors were designed and constructed to allow for stable replication and selection in our *S. meliloti* conjugative donor, as well as several conjugative recipient organisms including *E. coli*, *S. cerevisiae*, and *P. tricornutum*. These organisms were chosen as conjugative recipients as they are well-characterized model strains for bacterial, yeast and algal systems. These vectors were constructed with a bacterial artificial chromosome (BAC) and yeast artificial chromosome (YAC) backbone allowing for replication in *E. coli* and *S. cerevisiae* [25]. The YAC also allows replication in *P. tricornutum* [25,26]. The MHS vectors contain an origin of replication captured from the native megaplasmid of *S. meliloti*, pSymA (*repA2B2C2*, denoted pAGE vectors), or the pSymB chromid (*repA1B1C1*, denoted pBGE vectors). Selectable markers include spectinomycin (Sp), tetracycline (Tc), or kanamycin/neomycin (Km/Nm) resistance for *S. meliloti* (but also conferring some resistance in *E. coli*), nourseothricin N-acetyl transferase (Ntc) for *P. tricornutum* [27], HIS3 for *S. cerevisiae*, and chloramphenicol (Cm)

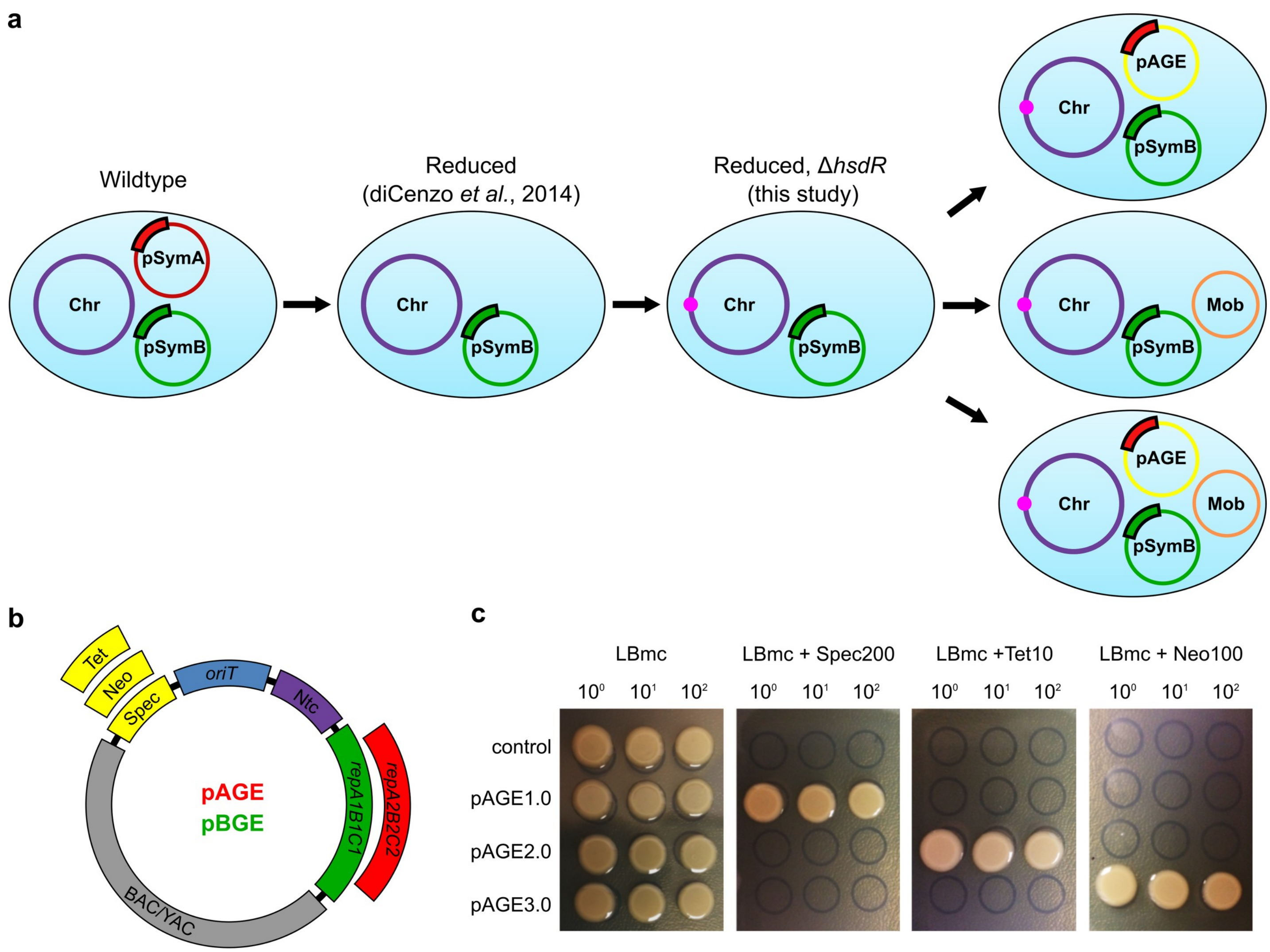

Fig 1. Development of designer *S. meliloti* strains and multi-functional vectors. (a) Schematic of the designer *S. meliloti* ΔpSymA Δ*hsdR* strains including the genome reduction and deletion of the restriction-system. Several versions of the designer strains were developed including strains with the pTA-Mob conjugative plasmid, and/or compatible genome engineering vectors (pAGE or pBGE) installed. (b) Vector map of pAGE/pBGE MHS vector with both standard and interchangeable components. Standard components include the BAC and YAC backbone (BAC/YAC), an origin of transfer (*oriT*), and the selectable marker for *P. tricornutum* nourseothricin N-acetyl transferase (Ntc). Interchangeable components include selectable markers for *S. meliloti*: tetracycline (Tet), kanamycin/neomycin (Neo), and spectinomycin (Spec); and origins of replication for *S. meliloti*: the pSymA origin (*repA2B2C2*), and pSymB origin (*repA1B1C1*). Three vectors (pAGE1.0, pAGE2.0, and pAGE3.0) were constructed utilizing the *repA2B2C2* origin with Spec, Tet or Neo selection, respectively. Three vectors (pBGE1.0, pBGE2.0, and pBGE3.0) were constructed utilizing the *repA1B1C1* origin with Spec, Tet or Neo selection, respectively. (c) Following conjugation of three pAGE plasmids from *E. coli* ECGE101 conjugative strain to *S. meliloti* RmP4122 *Δ*pSymA *ΔhsdR* Nm^s, an antibiotic selection test for each plasmid was performed. The pAGE1.0, pAGE2.0, and pAGE3.0 vectors in *S. meliloti* contain antibiotic resistance markers for Spec, Tet, and Neo, respectively. *S. meliloti* without a plasmid (control) was plated alongside *S. meliloti* harbouring pAGE1.0, pAGE2.0, or pAGE3.0 on LBmc agar, and LBmc agar supplemented with either spectinomycin (200 μg mL^{-1}), tetracycline (10 μg mL^{-1}), or neomycin (100 μg mL^{-1}).

https://doi.org/10.1371/journal.pone.0206781.g001

resistance for *E. coli*. Finally, all MHS vectors contain an origin of transfer (*oriT*) that is acted on by proteins encoded on the conjugative plasmid pTA-Mob, and is necessary for mobilization of the MHS vectors from *S. meliloti* to recipient organisms (Fig 1B). Three versions of the pAGE vector differing only in the *S. meliloti* selective marker, pAGE1.0 (Sp), pAGE2.0 (Tc), and pAGE3.0 (Km/Nm), were constructed with the pSymA origin (*repA2B2C2*). An additional three pBGE vectors differing only in the *S. meliloti* selective marker, pBGE1.0 (Sp), pBGE2.0 (Tc), and pBGE3.0 (Km/Nm), were constructed with the pSymB origin (*repA1B1C1*). Once

assembled [28], the vectors were transformed into *E. coli* Epi300 (Epicentre) cells, isolated and confirmed to be correctly assembled by a diagnostic restriction digest (Figure B in S1 File). The three pAGE vectors were conjugated to *S. meliloti* RmP4122 *Δ*pSymA *ΔhsdR* Nm^s from *E. coli* ECGE101 conjugative strains, and *S. meliloti* transconjugants were plated on their respective antibiotic selections. We demonstrated that pAGE1.0, pAGE2.0, and pAGE3.0 in *S. meliloti* provide resistance to Sp, Tc, and Nm, respectively (Fig 1C). Following transformation of pAGE2.0 into *S. meliloti* RmP4122 *Δ*pSymA *ΔhsdR* Nm^s, vector stability was tested by iterative subculturing every 10 generations to a total of about 50 generations. We observed, on average, that approximately 25% of vectors were lost after 50 generations, as determined by the number of colonies unable to grow on selective media (LBmc 38 μM $FeCl_3$ 2 μM $CoCl_2$ Tc 5 μg/mL) after restreaking from non-selective media (LBmc 38 μM $FeCl_3$ 2 μM $CoCl_2$) (Figure C in S1 File and Table A in S2 File).

Optimization of DNA transfer to *S. meliloti* via electroporation, a new polyethylene glycol transformation method and conjugation

In order to develop *S. meliloti* as a host, a highly efficient transformation method is required for the uptake of DNA. Currently, the most common transformation method used in *S. meliloti* is electroporation. Optimization of this method through transformation of *S. meliloti* RmP4122 *Δ*pSymA *ΔhsdR* Nm^s with three pAGE vectors (~18 kb) produced efficiencies averaging 1.4 x 10^5 CFU $μg^{-1}$ of DNA (Figure D in S1 File and Table B in S2 File). Additionally, since a PEG transformation method was successfully applied to move large DNA fragments (>1 Mb) in the transplantation protocol required to create the first synthetic cell [4], we developed a PEG-mediated transformation method in *S. meliloti* and were able to obtain efficiencies on average of 2.1 x 10^3 CFU $μg^{-1}$ of DNA (Figures E and F in S1 File and Table B in S2 File). In addition, conjugation has been previously established as a method of DNA transfer to *S. meliloti* [19,29], therefore we have developed an improved conjugation protocol from our conjugative *E. coli* ECGE101 strain carrying the pTA-Mob and pAGE1.0 vectors. Using this method we obtained a conjugation efficiency averaging 4 transconjugants per every 10 recipient cells (Table B in S2 File).

Direct DNA transfer (via conjugation) from *S. meliloti* to *E. coli*, *S. cerevisiae* and *P. tricornutum*

We utilized the designer *S. meliloti* RmP4098 ΔpSymA Δ*hsdR* host strain carrying the pTA--Mob conjugative plasmid and pAGE1.0 compatible genome engineering vector to develop direct DNA transfer of pAGE1.0 (via conjugation) from *S. meliloti* to *E. coli*, *S. cerevisiae*, and *P. tricornutum* (Fig 2A–2C and 2E). The ~18 kb pAGE1.0 vector contains the pSymA origin (*repA2B2C2*) and the spectinomycin selectable marker for *S. meliloti*. Conjugation efficiencies were calculated as the number of transconjugants per recipient cell from the *S. meliloti* conjugative donor to *E. coli*, *S. cerevisiae*, and *P. tricornutum* as 2.22 x 10^{-1}, 7.99 x 10^{-6} and 9.40 x 10^{-5}, respectively (Fig 3 and Table C in S2 File). Additionally, we have developed a high throughput conjugation protocol in a 96-well plate from *S. meliloti* to *P. tricornutum* that could be used for large-scale experiments and in an automated facility (Fig 2D and 2E). Development of these direct DNA transfer protocols is a critical step in the development of *S. meliloti* as a robust host for genome engineering and will facilitate its use in synthetic biology applications.

Next, we evaluated the conjugated pAGE1.0 vectors for DNA rearrangements and potential mutations that could have been introduced during conjugation from *S. meliloti* to *E. coli*, *S. cerevisiae* and *P. tricornutum*. In the first case, 20 *E. coli* transconjugant vectors were induced

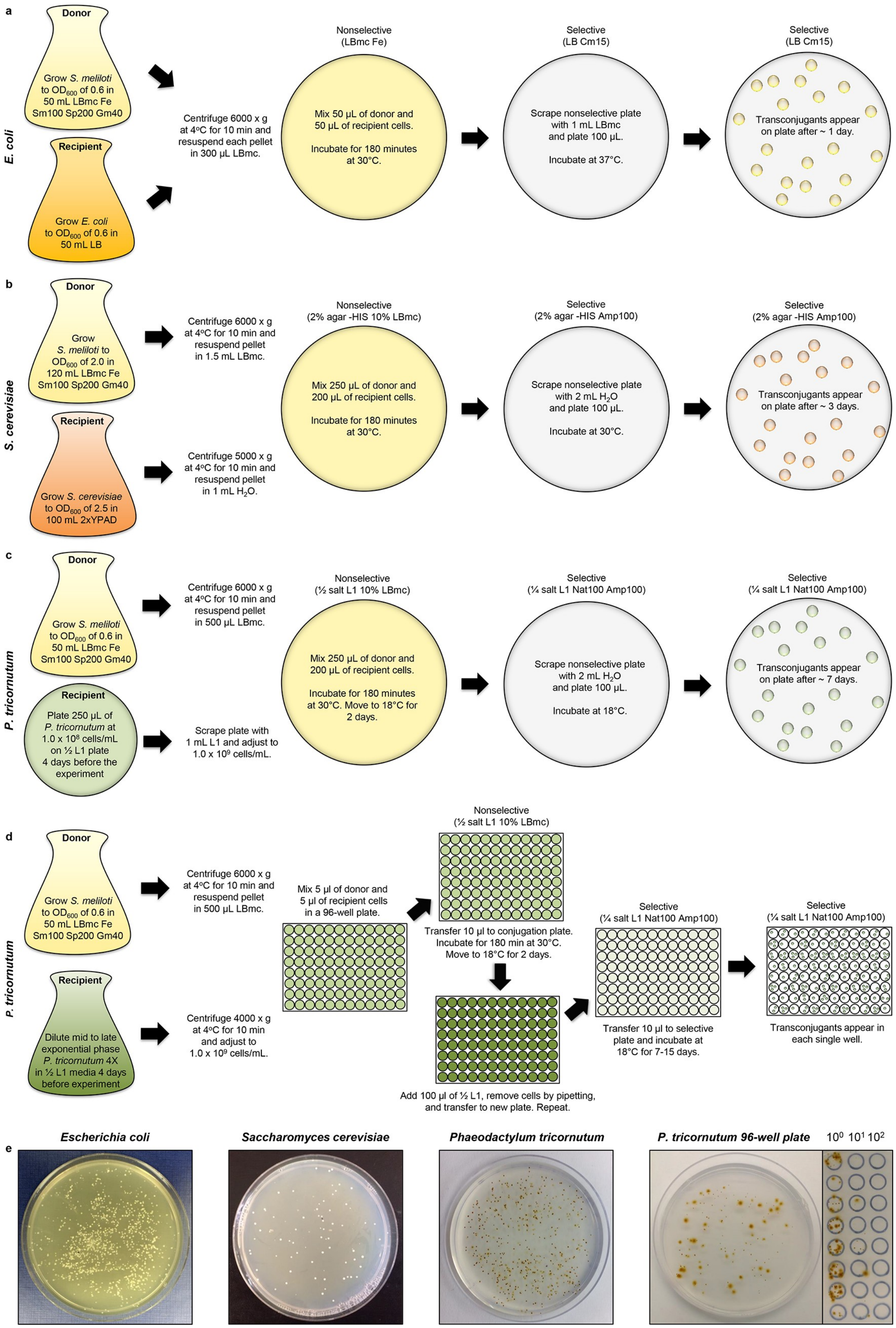
a
E. coli
Donor
Grow S. meliloti to OD_{600} of 0.6 in 50 mL LBmc Fe Sm100 Sp200 Gm40
Recipient
Grow E. coli to OD_{600} of 0.6 in 50 mL LB
Centrifuge 6000 x g at 4°C for 10 min and resuspend each pellet in 300 µL LBmc.
Nonselective (LBmc Fe)
Mix 50 µL of donor and 50 µL of recipient cells. Incubate for 180 minutes at 30°C.
Selective (LB Cm15)
Scrape nonselective plate with 1 mL LBmc and plate 100 µL. Incubate at 37°C.
Selective (LB Cm15)
Transconjugants appear on plate after ~ 1 day.
b
S. cerevisiae
Donor
Grow S. meliloti to OD_{600} of 2.0 in 120 mL LBmc Fe Sm100 Sp200 Gm40
Centrifuge 6000 x g at 4°C for 10 min and resuspend pellet in 1.5 mL LBmc.
Recipient
Grow S. cerevisiae to OD_{600} of 2.5 in 100 mL 2xYPAD
Centrifuge 5000 x g at 4°C for 10 min and resuspend pellet in 1 mL H_2O.
Nonselective (2% agar -HIS 10% LBmc)
Mix 250 µL of donor and 200 µL of recipient cells. Incubate for 180 minutes at 30°C.
Selective (2% agar -HIS Amp100)
Scrape nonselective plate with 2 mL H_2O and plate 100 µL. Incubate at 30°C.
Selective (2% agar -HIS Amp100)
Transconjugants appear on plate after ~ 3 days.
c
P. tricornutum
Donor
Grow S. meliloti to OD_{600} of 0.6 in 50 mL LBmc Fe Sm100 Sp200 Gm40
Centrifuge 6000 x g at 4°C for 10 min and resuspend pellet in 500 µL LBmc.
Recipient
Plate 250 µL of P. tricornutum at 1.0 x 10^8 cells/mL on ½ L1 plate 4 days before the experiment
Scrape plate with 1 mL L1 and adjust to 1.0 x 10^9 cells/mL.
Nonselective (½ salt L1 10% LBmc)
Mix 250 µL of donor and 200 µL of recipient cells. Incubate for 180 minutes at 30°C. Move to 18°C for 2 days.
Selective (¼ salt L1 Nat100 Amp100)
Scrape nonselective plate with 2 mL H_2O and plate 100 µL. Incubate at 18°C.
Selective (¼ salt L1 Nat100 Amp100)
Transconjugants appear on plate after ~ 7 days.
d
P. tricornutum
Donor
Grow S. meliloti to OD_{600} of 0.6 in 50 mL LBmc Fe Sm100 Sp200 Gm40
Centrifuge 6000 x g at 4°C for 10 min and resuspend pellet in 500 µL LBmc.
Recipient
Dilute mid to late exponential phase P. tricornutum 4X in ½ L1 media 4 days before experiment
Centrifuge 4000 x g at 4°C for 10 min and adjust to 1.0 x 10^9 cells/mL.
Mix 5 µl of donor and 5 µl of recipient cells in a 96-well plate.
Nonselective (½ salt L1 10% LBmc)
Transfer 10 µl to conjugation plate. Incubate for 180 min at 30°C. Move to 18°C for 2 days.
Add 100 µl of ½ L1, remove cells by pipetting, and transfer to new plate. Repeat.
Selective (¼ salt L1 Nat100 Amp100)
Transfer 10 µl to selective plate and incubate at 18°C for 7-15 days.
Selective (¼ salt L1 Nat100 Amp100)
Transconjugants appear in each single well.
e
Escherichia coli
Saccharomyces cerevisiae
Phaeodactylum tricornutum
P. tricornutum 96-well plate
10^0 10^1 10^2

Fig 2. Optimized conjugation protocols from *S. meliloti* to various recipient organisms. Antibiotic concentrations are given in μg mL^{-1} The *S. meliloti* RmP4098 ΔpSymA Δ*hsdR* strain used is resistant to streptomycin (100 μg mL^{-1}) and requires Fe (38 μM $FeCl_3$) supplementation for growth. The pAGE1.0 plasmid used confers resistance to spectinomycin (200 μg mL^{-1}). Media supplementation may vary with chosen *S. meliloti* host and MHS vector. (a) Schematic of protocol for conjugation of pAGE1.0 from *S. meliloti* to *E. coli*. (b) Schematic of protocol for conjugation of pAGE1.0 vector from *S. meliloti* to *S. cerevisiae*. (c) Schematic of protocol for conjugation of pAGE1.0 vector from *S. meliloti* to *P. tricornutum*–standard protocol. Note that ¼ salt L1 plates were made with ¼ aquil salts concentration (half that of typical ½ L1 plates), keeping all other components the same as in ½ L1 plates. (d) Schematic of protocol for conjugation of pAGE1.0 vector from *S. meliloti* to *P. tricornutum*– 96-well plate protocol. (e) Examples of plates containing transconjugants resulting from conjugation from *S. meliloti* to *E. coli*, *S. cerevisiae*, and *P. tricornutum* (standard and in a 96-well plate). *P. tricornutum* transconjugants from the 96-well plate protocol are shown with 100 uL plated on a full plate, and 10 uL spot plated and serially diluted.

https://doi.org/10.1371/journal.pone.0206781.g002

with 0.1% arabinose to obtain high copy number and were directly isolated from *E. coli*. The pAGE1.0 vector replicates as a low-copy plasmid in *S. cerevisiae* and *P. tricornutum* and it cannot be induced with arabinose to obtain high copy number within these species. Therefore, 60 vectors from *S. cerevisiae* and *P. tricornutum* transconjugants were isolated and transformed into *E. coli*, then induced and once again isolated to obtain high quality DNA. DNA isolated from 59 out of 60 *S. cerevisiae* transconjugants and 58 out of 60 *P. tricornutum* transconjugants resulted in *E. coli* colonies following transformation (Table D in S2 File). Then, 20 *E. coli* transformants (each transformed with DNA from independent *S. cerevisiae* colonies) and 30 *E. coli* transformants (each transformed with DNA from independent *P. tricornutum* colonies) were selected, induced with 0.1% arabinose, and the vector DNA was isolated. Diagnostic restriction digests were performed on these vectors using EcoRV-HF, and the number of vectors with the expected banding pattern was 18/20 originating from *E. coli*, 19/20 originating from *S. cerevisiae* and 16/30 originating from *P. tricornutum* (Figures G-I in S1 File and Table D in S2 File). Therefore, we can observe that correct pAGE1.0 vectors can be rescued when conjugated from *S. meliloti* 90% of time in *E. coli*, 93% of the time in *S. cerevisiae*, and 52% of the time in *P. tricornutum*. Finally, sequencing of pAGE1.0 plasmids recovered from *E. coli*, *S. cerevisiae* and *P.*

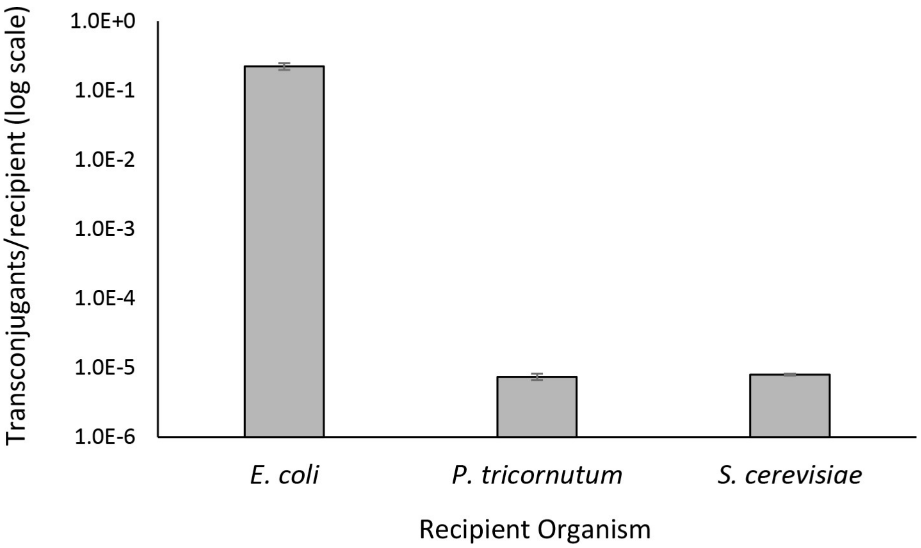

Fig 3. Conjugation efficiency of direct transfer of pAGE1.0 from *S. meliloti* donor to *E. coli*, *S. cerevisiae*, and *P. tricornutum* recipients. Conjugation efficiency for each donor-recipient pair. Post-conjugation non-selective plates and pAGE1.0 selective plates were used to determine the conjugation efficiency (calculated as transconjugants/recipient) between each donor and recipient pair. Efficiencies are represented as bar plots with three biological and three technical replicates, with error bars illustrating standard error of the mean.

https://doi.org/10.1371/journal.pone.0206781.g003

tricornutum transconjugants with a correct banding pattern following digestion with EcoRV-HF, revealed that no significant mutations were introduced.

In summary, pAGE and pBGE MHS vectors were developed and shown to be stably propagated in *S. meliloti*, *E. coli*, *S. cerevisiae*, and *P. tricornutum*. These multi-host and multi-functional vectors have promising applications for the cloning of large, high G+C content DNA fragments and their direct transfer to prokaryotic and eukaryotic target organisms. Conjugation of these vectors from *S. meliloti* to *E. coli*, *S. cerevisiae*, and *P. tricornutum*, as well as showing *S. meliloti* as a conjugative recipient, illustrates that *S. meliloti* can be a suitable host organism for inter-kingdom transfer of DNA. Due to the range of recipient organisms demonstrated, conjugation could be expanded to other target organisms in the future. These hosts will be invaluable for the cloning and installation of genes, synthetic chromosomes, or large biosynthetic pathways, the study of organisms lacking genetic tools, and specifically for applications in industrially and agriculturally important strains such as plants, marine organisms, and soil microbiomes.

Materials and methods

Microbial strains and growth conditions

Sinorhizobium meliloti strains were grown at 30˚C in LBmc medium (10 g tryptone, 5 g yeast extract, 5 g NaCl, 0.301 g $MgSO_4$, and 0.277 g anhydrous $CaCl_2$ were dissolved in sddH_2O to a final volume of 1 L, and then autoclaved. Solid plates contained 1.5% agar.) supplemented with appropriate antibiotics (streptomycin (100 μg mL^{-1}), spectinomycin (200 μg mL^{-1}), gentamicin (40 μg mL^{-1}), tetracycline (5 μg mL^{-1}), neomycin (100 μg mL^{-1})), and 38 μM $FeCl_3$ and/or 2 μM $CoCl_2$, when appropriate. *Saccharomyces cerevisiae* VL6−48 (ATCC MYA-3666: MATα his3-Δ200 trp1-Δ1 ura3−52 lys2 ade2−1 met14 cir^0) was grown at 30˚C in rich medium (2X YPD) supplemented with 80 mg L^{-1} adenine hemisulfate, or yeast synthetic complete medium lacking histidine supplemented with 60 mg L^{-1} adenine sulfate (Teknova, Inc.) [9]. *Escherichia coli* (Epi300, Epicenter) was grown at 37˚C in Luria Broth (LB) supplemented with appropriate antibiotics (chloramphenicol (15 μg mL^{-1})). *Escherichia coli* (ECGE101) was grown at 37˚C in Luria Broth (LB) supplemented with appropriate antibiotics (chloramphenicol (15 μg mL^{-1}), gentamicin (40 μg mL^{-1})), and diaminopimelic acid (60 μg mL^{-1}). *Phaeodactylum tricornutum* (Culture Collection of Algae and Protozoa CCAP 1055/1) was grown in L1 medium without silica at 18˚C under cool white fluorescent lights (75 μE m^{-2} s^{-1}) and a photoperiod of 16 h light:8 h dark [27]. L1 media was prepared as previously described [27].

Development of Δ*hsdR S. meliloti* strains

Designer *S. meliloti* strains used in this study were created by taking the strain RmP3500, which lacks pSymA and pSymB where the *engA-tRNA*^{Arg}*-rmlC* genomic region has been introduced into the chromosome [30] and re-introducing either pSymA or pSymB or both. Specifically, RmP4098, was made by re-introducing pSymB from RmP3491 into the strain RmP3910, which is derived from RmP3500, to create the strain RmP3950. From there, a Nm resistance cassette from strain RmP3975 was transduced into strain RmP3950. Double homologous recombination of the Nm cassette from RmP3975 into the genome of RmP3950 was selected on Nm Sm plates. The resulting strain, RmP4098, is ΔpSymA pSymB+ *hsdR*::Nm, Nm^R Sm^R.

RmP3975 is a strain where the *hsdR* restriction gene has been replaced by a Nm resistance cassette. This strain was created by first PCR amplifying a downstream region and upstream region of the *hsdR* gene and cloning these PCR products into the *XbaI* site of pUCP30T using SLiC [31], then transforming into chemically competent DH5α. This construct was then verified via sequencing using M13 universal primers. The pUCP30T plasmid (Gm^R) with the *hsdR*

upstream/downstream regions was then transformed into an *E. coli* strain harboring pKD46 (Amp^R) which includes genes for lambda red recombinase, the resulting strain was named M2453. A Km/Nm cassette for *hsdR* deletion was then PCR amplified using high-fidelity DNA polymerase and purified. The cassette consisted of the antibiotic resistance gene flanked by regions of homology to the *hsdR* regions cloned into pUCP30T. The cassette was then electroporated into competent M2453 cells (grown in 1mM Arabinose to induce lambda red recombinase genes) and selected using Km (25 μg mL^{-1}). Cells were then patched on Km (25 μg mL^{-1}), Gm (10 μg mL^{-1}) and Amp (100 μg mL^{-1}). A Km^R, Gm^R, and Amp^S strain was then streak purified and named M2459. The resulting vector was then conjugated into RmP110 as recipient and M2459 as donor. Selection was done in Sm (200 μg mL^{-1}) Nm (200 μg mL^{-1}) plates. Resulting transconjugants were then patched on Gm (10 μg mL^{-1}), (Nm 200 μg mL^{-1}) and Sm (200 μg mL^{-1}) plates and a Gm sensitive colony was streak purified (indicating double recombination of cassette with *hsdR* locus). The strain was then verified using diagnostic primers that spanned the *hsdR* upstream/downstream region with the Km/Nm cassette.

The Nm resistance cassette was then able to be transduced into the *S. meliloti* strains containing various combinations of pSymA and pSymB and selecting for Nm resistance. The Nm resistance cassette was then excised by introducing Flp recombinase to flip out the cassette using flanking FRT sites. The resulting strains are are RmP4258 (pSymA- pSymB- Δ*hsdR*), RmP4260 (pSymA+ pSymB- Δ*hsdR*), RmP4124 (pSymA- pSymB+ Δ*hsdR*), and RmP4125 (pSymA+ pSymB+ Δ*hsdR*).

Development of *E. coli* ECGE101 Δ*dapA* strain

The *dapA* gene replacement with lambda pir and an erythromycin cassette from strain βDH10B was replicated in Epi300, resulting in a strain containing *trfA* and requirement of diaminopimelic acid (DAP) supplementation for growth. This is useful because it allows for replication of plasmids with RK2 oriV and makes it a convenient donor in biparental conjugation because the DAP requirement eliminates the need for antibiotic counter-selection. A lambda red recombinase plasmid (pKD46) was electroporated into Epi300, because Epi300 is recA-. The *dapA* region from βDH10B was amplified using the flanking primers DAP1 and DAP2. The fragment was electroporated into *E. coli* Epi300 containing pKD46 and transformants were selected on LB with DAP (60 μg mL^{-1}) and erythromycin (200 μg mL^{-1}), and inability to grow in the absence of DAP was confirmed. Such transformants were cured of pKD46 by growing at 37˚C and confirming ampicillin (100 μg mL^{-1}) sensitivity.

Vector construction (pAGE/pBGE multi-host shuttle vectors)

Briefly, pAGE and pBGE vectors were constructed based on the pCC1BAC vector to which elements for replication in yeast were added. This backbone was amplified from plasmid p0521s (created at Venter institute and available on Addgene). This will allow replication and selection in yeast and *E. coli*. Additionally, it is low copy vector that can be induced to high copy with arabinose. Other components amplified for vector assembly include the antibiotic resistance cassettes for selection in *S. meliloti*, *repA2B2C2* or *repA1B1C1* for replication in *S. meliloti*, origin of transfer (*oriT*) for conjugation, and selective marker for algae (Ntc). The six fragments were PCR amplified and assembled in yeast using a yeast spheroplast transformation method. Next, DNA was isolated from yeast and moved to *E. coli* strain PG5alpha and genotyped using a multiplex PCR screen. Correct vectors were moved from PG5alpha to Epi300 and Epi300 pTA-Mob. Diagnostic digests were performed to confirm correct assembly of vectors.

Vector stability assay of pAGE2.0

To characterize the ability of the Δ*hsdR* strains to maintain these large pAGE vectors, stability assays were performed to determine how long pAGE2.0 can be maintained in RmP4122 *Δ*pSymA*Δ**hsdR*Nm^s. This was performed in triplicate. Single colonies harboring pAGE2.0 were inoculated into LBmc $FeCl_3$ + $CoCl_2$ with Tc (5μg mL^{-1}) at 30˚C. The next day, 100uL of culture diluted to 10^{-6} was plated on LBmc $FeCl_3$ + $CoCl_2$ and grown 3 days at 30˚C. Cultures were subcultured 1000X in LBmc $FeCl_3$ + $CoCl_2$ without antibiotics and grown overnight. Approximately 10 doublings occurred per day. The next day, cultures were diluted to 10^{-6} and again 100μL was plated on LBmc $FeCl_3$ + $CoCl_2$. Cultures were subcultured again as before. When visible, 100 colonies were patched onto LBmc $FeCl_3$ + $CoCl_2$ with Tc (5μg mL^{-1}) and without Tc as a control to ensure colony viability. These plates were incubated at 30˚C for 3 days. The number of patched colonies that were able to grow on selective media was then recorded. The experiment was performed for 5 days to assess stability of pAGE2.0.

Electroporation of *S. meliloti*

Preparation of competent *S. meliloti* cells for electroporation. Grow 500 mL of *S. meliloti* overnight in LBmc, 38 μM $FeCl_3$, and streptomycin 100 μg mL^{-1} at 30˚C with shaking incubation to an OD_{600} of 2.0. Incubate flask on ice for 10 min then pellet at 6000 x g at 4˚C for 10 min. Resuspend cells in 250 mL of sddH_2O by gentile agitated in a water bath, top up volume to 500 mL, then pellet with the same conditions. Repeat this wash step with sddH_2O two additional times, and then repeat once more with the same volume of 10% glycerol. Resuspend the pellet in 3 mL of 10% glycerol, flash freeze 200 μL aliquots and store at -80˚C.

Electroporation of *S. meliloti*. Incubate frozen cells on ice until fully thawed (about 15 min). Add 50 ng (in 1 μL) of DNA to 50 μL of competent cells in a 1.5 mL Eppendorf tube on ice, flick to mix, and incubate on ice for 5 min. Add transformation mixture to a 0.1 mm path length cuvette on ice and electroporate at 1.8 kV. Immediately add 1 mL LBmc 38μM $FeCl_3$ and recover in a test tube at 30˚C with shaking incubation (225rpm) for 120 min. Spread 500 μL of the transformation mixture on LBmc plates containing 38 μM $FeCl_3$, 100 μg mL^{-1} streptomycin, and an appropriate concentration of antibiotic selection based on the transformed DNA. Incubate plates at 30˚C for 3 days to allow for colony formation.

PEG-mediated transformation of *S. meliloti*

Preparation of competent *S. meliloti* cells for PEG-mediated transformation. *S. meliloti* cells were cultured overnight in LBmc, 38μM $FeCl_3$, and 100 μg mL^{-1} streptomycin at 30˚C with shaking incubation (225rpm). Upon reaching OD_{600} = 0.4, cells were harvested into sterile 500 mL centrifuge bottles, incubated on ice for 10 minutes, and pelleted at 4000 x g and 4˚C for 10 minutes. Cells were then resuspended in 100 mL of ice-cold 100 mM $CaCl_2$ by gentle pipetting and incubated on ice for an additional 30 minutes. Following incubation, cells were pelleted again at 4000 x g and 4˚C for 10 minutes and resuspended in 1.25 mL of ice-cold 100 mM $CaCl_2$ + 15% glycerol. The final resuspension volume was split into 25 μL aliquots, flash frozen using liquid nitrogen, and stored at -80˚C for later use.

PEG-mediated transformation of *S. meliloti*. Frozen cells (25 μL aliquots) were incubated on ice until fully thawed. Next, 200 ng (in 3 μL) of supercoiled pAGE DNA and 25 μL of 10% PEG 4000 were then added to the reaction tube and mixed evenly by gentle pipetting. Cells were then incubated on ice for 30 minutes, transferred to a 40˚C water bath for 8 minutes, and immediately placed back on ice for 10 minutes. Following, 500 μL of LBmc, 38μM $FeCl_3$ was added to the reaction tube and cells were recovered at 30˚C with shaking incubation (225rpm) for 90 minutes. Following recovery, 250 μL of the transformation mixture were

spread on LBmc plates containing 38 μM $FeCl_3$, 100 μg mL^{-1} streptomycin, and an appropriate concentration of antibiotic selection. Plates were incubated at 30˚C for 3 days to allow for colony formation.

Transfer of DNA from *E. coli* to *S. meliloti* via conjugation

Preparation of *S. meliloti* (RmP4122) cells. An overnight culture (OD_{600} = 2.0) in LBmc, 38μM $FeCl_3$, streptomycin 100 μg mL^{-1} was diluted 20x to make 20 mL culture and grown 6 hours shaking at 30˚C in LBmc supplemented with streptomycin 100 μg mL^{-1} and Fe to OD_{600} of 0.9. The culture was diluted 2000X and grown with shaking at 30˚C in 50 mL LBmc supplemented with streptomycin 100 μg mL^{-1} and Fe to OD_{600} of 0.6. The culture was centrifuged for 10 min at 6,000 x g at 4˚C and resuspended in 300 μL of LBmc media.

Preparation of *E. coli* (ECGE 101 pTA-Mob pAGE1.0) cells. Saturated overnight culture of *E. coli* was diluted 20X into 50 mL LB supplemented with diaminopimelic acid 60 μg mL^{-1}, chloramphenicol 15 μg mL^{-1}, and gentamicin 20 μg mL^{-1} and grown with shaking at 37˚C to OD_{600} of 0.6. The culture was centrifuged for 10 min at 6,000 x g at 4˚C and resuspended in 300 μL of LBmc media.

Conjugation from *E. coli* to *S. meliloti*. First, 50 μL of *E. coli* cells and 50 μL of *S. meliloti* cells were mixed directly on LBmc plates supplemented with 38μM $FeCl_3$ and diaminopimelic acid 60 μg mL^{-1} and incubated for 180 minutes at 30˚C. Then, 1 mL of LBmc media was added to plates, cells were scraped, and 100 μL (from a dilution series of 10^{-3} to 10^{-9}) was plated on LBmc plates supplemented with Fe, streptomycin 100 μg mL^{-1}, and spectinomycin 200 μg mL^{-1} (Note: plates should be at least 35 mL thick). Plates were incubated at 30˚C for 3 days before colonies were counted.

Transfer of DNA from *S. meliloti* to *E. coli* via conjugation

Preparation of *S. meliloti* (RmP4098 pTA-Mob pAGE1.0) cells. Stock culture (OD_{600} = 2.0) was diluted 20X to make 20 mL culture and grown 6 hours shaking at 30˚C in LBmc supplemented with streptomycin 100 μg mL^{-1} spectinomycin 200 μg mL^{-1}, gentamicin 40 μg mL^{-1} and Fe to OD_{600} of 0.3. The culture was diluted 500X and grown with shaking at 30˚C in 50 mL LBmc supplemented with streptomycin 100 μg mL^{-1}, spectinomycin 200 μg mL^{-1}, gentamicin 40 μg mL^{-1} to OD_{600} of 0.6. The culture was centrifuged for 10 min at 6,000 x g at 4˚C and resuspended in 300 μL of LBmc media.

Preparation of *E. coli* (Epi300) cells. Saturated overnight culture of *E. coli* was diluted 20X into 50 mL LB and grown with shaking at 37˚C to OD_{600} of 0.6. The culture was centrifuged for 10 min at 6,000 x g at 4˚C and resuspended in 300 μL of LBmc media.

Conjugation from *S. meliloti* to *E. coli*. First, 50 μL of *E. coli* cells and 50 μL of *S. meliloti* cells were mixed directly on LBmc plates supplemented with Fe and incubated for 180 minutes at 30˚C. Then, 1 mL of LBmc media was added to plates, cells were scraped, and 100 μL (from a dilution series of 10^{-3} to 10^{-9}) was plated on LB plates supplemented with chloramphenicol 15 μg mL^{-1}. Plates were incubated at 37˚C for 16 hours before colonies are counted.

Transfer of DNA from *S. meliloti* to *S. cerevisiae* via conjugation

Preparation of *S. meliloti* (RmP4098 pTA-Mob pAGE1.0) cells. Stock culture (OD_{600} = 2.0) was diluted 20x to make 20 mL culture and grown 6 hours shaking at 30˚C in LBmc supplemented with streptomycin 100 μg mL^{-1}, spectinomycin 200 μg mL^{-1}, gentamicin 40 μg mL^{-1} and 38μM $FeCl_3$ to OD_{600} of 0.3. The culture was diluted and grown with shaking at 30˚C in 120 mL LBmc supplemented with streptomycin 100 μg mL^{-1}, spectinomycin 200 μg mL^{-1}, gentamicin 40 μg mL^{-1}, acetosyringone 100 μg mL^{-1} to OD_{600} of 2.0 (arabinose was added to final

concentration of 100 μg mL^{-1} to the growing culture 1 hour before the target OD was reached). The culture was centrifuged for 10 min at 6,000 x g at 4˚C and resuspended in 1.5 mL of LBmc media.

Note: For simplicity, acetosyringone and arabinose steps can be removed from the final protocol without any substantial decrease in conjugation efficiency.

Preparation of *S. cerevisiae* (VL6-48) cells. *S. cerevisiae* was grown with shaking at 30˚C in 100 mL of 2X YPAD media to OD_{600} of 2.5. The culture was centrifuged for 10 min at 5,000 x g and resuspended in 1 ml of H_20.

Conjugation from *S. meliloti* to *S. cerevisiae*. First, 200 μL of *S. cerevisiae* cells and 250 μL of *S. meliloti* was directly mixed on a 2% -HIS plate supplemented with 10% LBmc, 38μM $FeCl_3$ and acetosyringone 100 μg mL^{-1} (Note: plates were dried out in the hood for 1 hour prior to conjugation). Then plates were incubated for 180 minutes at 30˚C. Then, 2 mL of $sddH_20$ was added to plates and cells were scraped. Next, 100 μL of the scraped cells was plated on 2% -HIS plates supplemented with ampicillin 100 μg mL^{-1}. Plates were incubated at 30˚C where colonies start to appear after 2–3 and colonies are counted after 5 days.

Note: For simplicity, accetosyringone steps can be removed from the final protocol without any substantial decrease in conjugation efficiency.

Transfer of DNA from *S. meliloti* to *P. tricornutum* via conjugation

Preparation of *P. tricornutum* cells. First, 250 μL of liquid grown culture was adjusted to 1.0 x 10^8 cells mL^{-1} using counts from a hemocytometer, was plated on ½L1 1% agar plates and grown for 4 days. Then, 1 mL of L1 media was added to the plate and cells were scraped, counted using a hemocytometer, and adjusted to a concentration of 1 x 10^9 cells mL^{-1}.

Preparation of *S. meliloti* (strain A-R-, pAGE1.0, pTA-Mob) cells. Stock culture (OD_{600} = 2.0) was diluted 20x to make 20 mL culture and grown 6 hours shaking at 30˚C in LBmc supplemented with spectinomycin 200 μg mL^{-1}, gentamicin 40 μg mL^{-1} and Fe to OD_{600} of 0.3. The culture was diluted 25X and grown for 12 hours with shaking at 30˚C in 50 mL LBmc supplemented with spectinomycin 200 μg mL^{-1}, gentamicin 40 μg mL^{-1} to OD_{600} of 0.6. The culture was centrifuged for 10 min at 5,000 x g at 4˚C and resuspended in 500 μL of LBmc media.

Conjugation from *S. meliloti* to *P. tricornutum*. First, 200 μL of *P. tricornutum* cells and 200 μL of *S. meliloti* cells were mixed directly on ½L1 10% LBmc 1% agar plates (Note: plates are dried in the biosafety cabinet for one hour before conjugation) and incubated for 180 minutes at 30˚C in the dark, then moved to 18˚C in the light and grown for 2 days. After two days, 2 mL of L1 media was added to plates, cells were scraped, and 100 μL (5%) was plated on ¼L1 1% agar plates supplemented with nourseothricin 100 μg mL^{-1}, and ampicillin 100 μg mL^{-1} (Note: plates should be at least 35 mL thick). Plates were incubated at 18˚C in the light/dark cycle and colonies start to appear after 7 days and are allowed to develop to 14 days before colonies are counted.

Transfer of DNA from *S. meliloti* to *P. tricornutum* via conjugation in a 96-well plate

Preparation of *P. tricornutum* cells. First, 200 μL of liquid grown culture was diluted using counts from a hemocytometer, and grown in ½L1 media for 4 days. Cell counts from a hemocytometer were used and culture was pelleted at 4000 x g 10 min 4˚C, and adjusted to a concentration of 1 x 10^9 cells mL^{-1}.

Preparation of *S. meliloti* (strain A-R-, pAGE1.0, pTA-Mob) cells. Stock culture (OD_{600} = 2.0) was diluted 20x to make 20 mL culture and grown 6 hours shaking at 30˚C in LBmc supplemented with spectinomycin 200 μg mL^{-1}, gentamicin 40 μg mL^{-1} and Fe to OD_{600} of 0.3.

The culture was diluted 25X and grown for 12 hours with shaking at 30˚C in 50 mL LBmc supplemented with spectinomycin 200 μg mL^{-1}, gentamicin 40 μg mL^{-1} to OD_{600} of 0.6. The culture was centrifuged for 10 min at 5,000 x g at 4˚C and resuspended in 500 μL of LBmc media.

Conjugation from *S. meliloti* to *P. tricornutum*. First, 5 μL of *P. tricornutum* cells and 5 μL of *S. meliloti* cells were mixed together in a 96-well plate. The mixture (10 μL) was transferred to a 96-well plate containing 200 μL of ½L1 10% LBmc 1% agar (note: plates are dried in the biosafety cabinet for one hour before conjugation). This conjugation plate was incubated for 180 minutes at 30˚C in the dark, then moved to 18˚C in the light and grown for 2 days. After two days, 100 μL of L1 media was added to wells and cells were scraped (X2), and 10 μL (5%) was plated on ¼L1 1% agar supplemented with nourseothricin 100 μg mL^{-1}, and ampicillin 100 μg mL^{-1} in a 96-well plate. Plates were incubated at 25˚C in the light/dark cycle for 24 hours and then 18˚C in the light/dark cycle for an additional 24 hours. Colonies start to appear after 7 days and are allowed to develop up to 14 days before colonies are counted.

Plasmid DNA isolation

Plasmid DNA (<60 kb) was isolated from *E. coli* using the BioBasic EZ-10 miniprep kit. Plasmid DNA was isolated from all other species using a modified alkaline lysis protocol. Plasmid DNA was isolated from all other species and *E. coli* containing plasmids >60 kb using the modified alkaline lysis protocol described below. Steps 1–3 are variable depending on the species, while steps 4–10 are common for all species. Steps 1–3 for *E. coli* and *S. meliloti*. (1) Five mL cultures were grown to saturation overnight. (2) Cells were pelleted at 5000 x g for 10 min at 4˚C, and the supernatant was discarded. (3) Cells were resuspended in 250 uL of resuspension buffer (which contained 240 ml P1 (Qiagen), 5 ml of 1.4 M b-Mercaptoethanol and 5 ml Zymolyase solution (Zymolyase solution: 200 mg Zymolyase 20 T (USB), 9 ml H2O, 1 ml 1 M Tris pH7.5, 10 ml 50% glycerol, stored at 20˚C). Steps 1–3 for *S. cerevisiae*. (1) Five mL of culture was grown to saturation. (2) Cells were pelleted at 5000 x g for 10 min 10 min at 4˚C, and the supernatant was discarded. (3) Cells were resuspended in 250 mL resuspension buffer (as described above) and incubated at 37˚C for 60 min. Steps 1–3 for *P. tricornutum*. (1) Five mL cultures were harvested during exponential growth phase. (2) Cells were pelleted at 4,000g for 10 min at 4˚C, and the supernatant was discarded. (3) Cells were resuspended in 250 mL resuspension buffer, which contained 235 ml P1 (Qiagen), 5 ml hemicellulase 100 mg ml 1, 5 ml of lysozyme 25 mg ml 1, and 5 ml Zymolyase solution (Zymolyase solution: 200 mg Zymolyase 20T (USB), 9 ml H2O, 1 ml 1 M Tris pH7.5, 10 ml 50% glycerol, stored at 20˚C) and then cells were incubated at 37˚C for 30 min. Steps 4–10 common for all species. (4) 250 uL of lysis buffer P2 (Qiagen) was added and samples were inverted 5–10 times to mix. (5) 250 ml of neutralization buffer P3 was added and samples were inverted 5–10 times to mix. (6) Then samples were spun down at 16,000g, 10 min at 4˚C (7) Supernatant was transferred to a clean tube and 750 ul ice-cold isopropanol was added and the samples were mixed by inversion and spun down at 16,000g, 10 min at 4˚C (8) Next the supernatant was removed and 750 ul ice-cold 70% EtOH was added and samples were mixed by inversion and spun down at 16,000g, 5 min. (9) Next the supernatant was discarded, pellets were briefly dried and resuspended in 50 uL of TE buffer. (10) After that the samples were kept at 37˚C for 30–60 min to dissolve.

Supporting information

S1 File. Supporting figures. Figure A. Verification of *hsdR* deletion in reduced *S. meliloti* strains by diagnostic PCR. Figure B. Diagnostic restriction digest of pAGE and pBGE vectors. Figure C. Vector stability assay of pAGE2.0 in *S. meliloti* over 50 generations. Figure D. Workflow of optimized electroporation transformation protocol for *S. meliloti*. Figure E. Workflow

of optimized PEG-mediated transformation protocol for *S*. *meliloti*. Figure F. PEG-mediated transformation of pAGE vectors into *S*. *meliloti*. Figure G. EcoRV-HF diagnostic digest of pAGE1.0 vectors extracted from 20 *E*. *coli* colonies following conjugation from *S*. *meliloti* to *E*. *coli*. Figure H. EcoRV-HF diagnostic digest of pAGE1.0 vectors extracted from 20 *E*. *coli* colonies following conjugation from *S*. *meliloti* to *P*. *tricornutum*. Figure I. EcoRV-HF diagnostic digest of pAGE1.0 vectors extracted from 20 *E*. *coli* colonies following conjugation from *S*. *meliloti* to *S*. *cerevisiae*.
(DOCX)

S2 File. Supporting tables. Table A. Vector stability of pAGE2.0 in *S*. *meliloti* RmP4122 ΔpSymA Δ*hsdR*. Table B. Efficiency of DNA transfer methods to *S*. *meliloti* RmP4122 ΔpSymA Δ*hsdR* including PEG-mediated transformation, electroporation and conjugation of pAGE vectors. Table C. Efficiency of pAGE1.0 conjugation from *S*. *meliloti* to recipient organisms and ratio of donor to recipient cells involved. Table D. Analysis of pAGE1.0 vectors recovered from conjugations from *S*. *meliloti* to *E*. *coli*, *S*. *cerevisiae*, and *P*. *tricornutum*. Table E. List of oligonucleotides used in this study. Table F. List of strains used in this study. Table G. List of vectors used in this study.
(DOCX)

Acknowledgments

This work was supported by a Genome Canada Grant (OGI-111) to T.C.C., T.M.F. and B.J.K. Research in the labs of D.R.E., T.C.C., T.M.F. and B.J.K. is also supported by Natural Sciences and Engineering Research Council of Canada (NSERC), RGPIN-2015-04800, RGPIN-2018-04754, RGPIN-2018-06481, RGPIN-2018-06172 respectively. In addition, this study was supported by Designer Microbes Inc (DMI). The funder provided support in the form of salaries for R.S.M., and P.J., and the specific role of these authors are articulated in the 'author contributions' section.

Author Contributions

Conceptualization: Trevor C. Charles, Turlough M. Finan, Bogumil J. Karas.

Data curation: Stephanie L. Brumwell, Michael R. MacLeod, Tony Huang, Ryan R. Cochrane, Preetam Janakirama.

Formal analysis: Stephanie L. Brumwell, Michael R. MacLeod, Tony Huang, Ryan R. Cochrane, Rebecca S. Meaney, Preetam Janakirama, Trevor C. Charles, Turlough M. Finan, Bogumil J. Karas.

Funding acquisition: Trevor C. Charles, Turlough M. Finan, Bogumil J. Karas.

Investigation: Stephanie L. Brumwell, Michael R. MacLeod, Tony Huang, Ryan R. Cochrane, Rebecca S. Meaney, Maryam Zamani, Ola Matysiakiewicz, Kaitlyn N. Dan, Preetam Janakirama, Trevor C. Charles, Bogumil J. Karas.

Methodology: Stephanie L. Brumwell, Michael R. MacLeod, Tony Huang, Ryan R. Cochrane, Rebecca S. Meaney, Ola Matysiakiewicz, Preetam Janakirama, Bogumil J. Karas.

Project administration: Turlough M. Finan.

Resources: David R. Edgell, Trevor C. Charles, Turlough M. Finan, Bogumil J. Karas.

Supervision: Preetam Janakirama, David R. Edgell, Trevor C. Charles, Turlough M. Finan, Bogumil J. Karas.

Validation: Stephanie L. Brumwell, Michael R. MacLeod, Tony Huang, Ryan R. Cochrane, Preetam Janakirama, Bogumil J. Karas.

Visualization: Stephanie L. Brumwell, Michael R. MacLeod, Tony Huang, Ryan R. Cochrane, Preetam Janakirama, Bogumil J. Karas.

Writing – original draft: Stephanie L. Brumwell, Michael R. MacLeod, Tony Huang, Preetam Janakirama, David R. Edgell, Trevor C. Charles, Turlough M. Finan, Bogumil J. Karas.

Writing – review & editing: Stephanie L. Brumwell, Michael R. MacLeod, Tony Huang, Ryan R. Cochrane, Rebecca S. Meaney, Preetam Janakirama, David R. Edgell, Trevor C. Charles, Turlough M. Finan, Bogumil J. Karas.

References

1. Rahn-Lee L, Byrne ME, Zhang M, Le Sage D, Glenn DR, Milbourne T, et al. A Genetic Strategy for Probing the Functional Diversity of Magnetosome Formation. PLoS Genet. 2015; 11: e1004811. https://doi.org/10.1371/journal.pgen.1004811 PMID: 25569806
2. Waller MC, Bober JR, Nair NU, Beisel CL. Toward a genetic tool development pipeline for host-associated bacteria. Curr Opin Microbiol. 2017; 38: 156–164. https://doi.org/10.1016/j.mib.2017.05.006 PMID: 28624690
3. Benders GA, Noskov VN, Denisova EA, Lartigue C, Gibson DG, Assad-Garcia N, et al. Cloning whole bacterial genomes in yeast. Nucleic Acids Res. 2010; 38: 2558–2569. https://doi.org/10.1093/nar/gkq119 PMID: 20211840
4. Gibson DG, Glass JI, Lartigue C, Noskov VN, Chuang R-Y, Algire MA, et al. Creation of a bacterial cell controlled by a chemically synthesized genome. Science. American Association for the Advancement of Science; 2010; 329: 52–56. https://doi.org/10.1126/science.1190719 PMID: 20488990
5. Hutchison CA, Chuang R-Y, Noskov VN, Assad-Garcia N, Deerinck TJ, Ellisman MH, et al. Design and synthesis of a minimal bacterial genome. Science (80-). American Association for the Advancement of Science; 2016; 351: aad6253. https://doi.org/10.1126/SCIENCE.AAD6253 PMID: 27013737
6. Benders GA, Noskov VN, Denisova EA, Lartigue C, Gibson DG, Assad-Garcia N, et al. Cloning whole bacterial genomes in yeast. Nucleic Acids Res. Oxford University Press; 2010; 38: 2558–2569. https://doi.org/10.1093/nar/gkq119 PMID: 20211840
7. Karas BJ, Tagwerker C, Yonemoto IT, Hutchison CA, Smith HO. Cloning the *Acholeplasma laidlawii* PG-8A Genome in *Saccharomyces cerevisiae* as a Yeast Centromeric Plasmid. ACS Synth Biol. American Chemical Society; 2012; 1: 22–28. https://doi.org/10.1021/sb200013j PMID: 23651007
8. Tagwerker C, Dupont CL, Karas BJ, Ma L, Chuang R-Y, Benders GA, et al. Sequence analysis of a complete 1.66 Mb Prochlorococcus marinus MED4 genome cloned in yeast. Nucleic Acids Res. Narnia; 2012; 40: 10375–10383. https://doi.org/10.1093/nar/gks823 PMID: 22941652
9. Karas BJ, Jablanovic J, Sun L, Ma L, Goldgof GM, Stam J, et al. Direct transfer of whole genomes from bacteria to yeast. Nat Methods. NIH Public Access; 2013; 10: 410–412. https://doi.org/10.1038/nmeth.2433 PMID: 23542886
10. Karas BJ, Moreau NG, Deerinck TJ, Gibson DG, Venter JC, Smith HO, et al. Direct Transfer of a Mycoplasma mycoides Genome to Yeast Is Enhanced by Removal of the Mycoides Glycerol Uptake Factor Gene glpF. ACS Synthetic Biology. American Chemical Society; 2019: 239–244. https://doi.org/10.1021/acssynbio.8b00449 PMID: 30645947
11. Noskov VN, Karas BJ, Young L, Chuang RY, Gibson DG, Lin YC, et al. Assembly of large, high G+C bacterial DNA fragments in yeast. ACS Synth Biol. 2012; 1: 267–273. https://doi.org/10.1021/sb3000194 PMID: 23651249
12. Karas BJ, Molparia B, Jablanovic J, Hermann WJ, Lin YC, Dupont CL, et al. Assembly of eukaryotic algal chromosomes in yeast. J Biol Eng. 2013; 7: 30. https://doi.org/10.1186/1754-1611-7-30 PMID: 24325901
13. Karas BJ, Suzuki Y, Weyman PD. Strategies for cloning and manipulating natural and synthetic chromosomes. Chromosom Res. 2015; 23: 57–68. https://doi.org/10.1007/s10577-014-9455-3 PMID: 25596826
14. Wright F, Bibb MJ. Codon usage in the G+C-rich Streptomyces genome. Gene. 1992; 113: 55–65. https://doi.org/10.1016/0378-1119(92)90669-G PMID: 1563633

15. Galardini M, Pini F, Bazzicalupo M, Biondi EG, Mengoni A. Replicon-dependent bacterial genome evolution: The case of Sinorhizobium meliloti. Genome Biol Evol. 2013; 5: 542–558. https://doi.org/10.1093/gbe/evt027 PMID: 23431003
16. diCenzo GC, MacLean AM, Milunovic B, Golding GB, Finan TM. Examination of Prokaryotic Multipartite Genome Evolution through Experimental Genome Reduction. PLoS Genet. 2014; 10: e1004742. https://doi.org/10.1371/journal.pgen.1004742 PMID: 25340565
17. diCenzo G, Milunovic B, Cheng J, Finan TM. The tRNAarg gene and engA are essential genes on the 1.7-Mb pSymB megaplasmid of Sinorhizobium meliloti and were translocated together from the chromosome in an ancestral strain. J Bacteriol. American Society for Microbiology; 2013; 195: 202–212. https://doi.org/10.1128/JB.01758-12 PMID: 23123907
18. MacLellan SR, Zaheer R, Sartor AL, MacLean AM, Finan TM. Identification of a megaplasmid centromere reveals genetic structural diversity within the repABC family of basic replicons. Mol Microbiol. 2006; 59: 1559–1575. https://doi.org/10.1111/j.1365-2958.2006.05040.x PMID: 16468995
19. Döhlemann J, Wagner M, Happel C, Carrillo M, Sobetzko P, Erb TJ, et al. A Family of Single Copy repABC -Type Shuttle Vectors Stably Maintained in the Alpha-Proteobacterium Sinorhizobium meliloti. ACS Synth Biol. American Chemical Society; 2017; 6: 968–984. https://doi.org/10.1021/acssynbio.6b00320 PMID: 28264559
20. Galibert F, Finan TM, Long SR, Pühler A, Abola P, Ampe F, et al. The composite genome of the legume symbiont Sinorhizobium meliloti. Science (80-). 2001; 293: 668–672. https://doi.org/10.1126/science.1060966 PMID: 11474104
21. Brothaerts W, Mitchell HJ, Weir B, Kaines S, Smith LMA, Yang W, et al. Gene transfer to plants by diverse species of bacteria. Nature. 2005; 433: 629–633. https://doi.org/10.1038/nature03309 PMID: 15703747
22. Strand TA, Lale R, Degnes KF, Lando M, Valla S. A new and improved host-independent plasmid system for RK2-based conjugal transfer. PLoS One. 2014; 9: 1–6. https://doi.org/10.1371/journal.pone.0090372 PMID: 24595202
23. Ferri L, Gori A, Biondi EG, Mengoni A, Bazzicalupo M. Plasmid electroporation of Sinorhizobium strains: The role of the restriction gene hsdR in type strain Rm1021. Plasmid. Elsevier Inc.; 2010; 63: 128–135. https://doi.org/10.1016/j.plasmid.2010.01.001 PMID: 20097223
24. Gerdes S, Scholle MD, Campbell JW, Balázsi G, Ravasz E, Daugherty MD, et al. Experimental determination and system level analysis of essential genes in Escherichia coli MG1655. J Bacteriol. 2003; 185: 5673–5684. https://doi.org/10.1128/JB.185.19.5673-5684.2003 PMID: 13129938
25. Karas BJ, Diner RE, Lefebvre SC, McQuaid J, Phillips APR, Noddings CM, et al. Designer diatom episomes delivered by bacterial conjugation. Nat Commun. Nature Publishing Group; 2015; 6. https://doi.org/10.1038/ncomms7925 PMID: 25897682
26. Diner RE, Noddings CM, Lian NC, Kang AK, McQuaid JB, Jablanovic J, et al. Diatom centromeres suggest a mechanism for nuclear DNA acquisition. Proc Natl Acad Sci U S A. National Academy of Sciences; 2017; 114: E6015–E6024. https://doi.org/10.1073/pnas.1700764114 PMID: 28673987
27. Slattery SS, Diamond A, Wang H, Therrien JA, Lant JT, Jazey T, et al. An Expanded Plasmid-Based Genetic Toolbox Enables Cas9 Genome Editing and Stable Maintenance of Synthetic Pathways in Phaeodactylum tricornutum. ACS Synth Biol. 2018; 7: 328–338. https://doi.org/10.1021/acssynbio.7b00191 PMID: 29298053
28. Kouprina N, Larionov V. Selective isolation of genomic loci from complex genomes by transformation-associated recombination cloning in the yeast Saccharomyces cerevisiae. Nat Protoc. 2008; 3: 371–377. https://doi.org/10.1038/nprot.2008.5 PMID: 18323808
29. House BL, Mortimer MW, Kahn ML. New recombination methods for Sinorhizobium meliloti genetics. Appl Environ Microbiol. 2004; 70: 2806–2815. https://doi.org/10.1128/AEM.70.5.2806-2815.2004 PMID: 15128536
30. diCenzo GC, Zamani M, Milunovic B, Finan TM. Genomic resources for identification of the minimal N2-fixing symbiotic genome. Environ Microbiol. 2016; 18: 2534–2547. https://doi.org/10.1111/1462-2920.13221 PMID: 26768651
31. Jeong JY, Yim HS, Ryu JY, Lee HS, Lee JH, Seen DS, et al. One-step sequence-and ligation-independent cloning as a rapid and versatile cloning method for functional genomics studies. Appl Environ Microbiol. 2012; 78: 5440–5443. https://doi.org/10.1128/AEM.00844-12 PMID: 22610439
